# Characterization of a *cdc14* null allele in *Drosophila melanogaster*

**DOI:** 10.1242/bio.035394

**Published:** 2018-06-26

**Authors:** Leif R. Neitzel, Matthew R. Broadus, Nailing Zhang, Leah Sawyer, Heather A. Wallace, Julie A. Merkle, Jeanne N. Jodoin, Poojitha Sitaram, Emily E. Crispi, William Rork, Laura A. Lee, Duojia Pan, Kathleen L. Gould, Andrea Page-McCaw, Ethan Lee

**Affiliations:** 1 Department of Cell and Developmental Biology, Vanderbilt University Medical Center, Nashville, TN 37232, USA; 2 Program in Developmental Biology, Vanderbilt University School of Medicine, Nashville, TN 37232, USA; 3 Department of Cell Biology, Harvard Medical School, Boston, MA 02115, USA; 4 Department of Physiology, University of Texas Southwestern Medical Center, Dallas, TX 75390-9040, USA; 5 Division of Genetics, Brigham and Women's Hospital, Boston, MA 02115, USA; 6 Department of Genetics, Harvard Medical School, Boston, MA 02115, USA; 7 Department of Molecular Biology, Princeton University, Princeton, NJ 08544, USA; 8 Department of Biology, Massachusetts Institute of Technology, Cambridge, MA 02142, USA; 9 Department of Microbiology, New York University Langone Medical Center, New York, NY 10016, USA; 10 Department of Pathology, Microbiology, and Immunology, Vanderbilt University Medical Center, Nashville, TN 37232, USA; 11 Vanderbilt Ingram Cancer Center, Vanderbilt University Medical Center, Nashville, TN 37232, USA

**Keywords:** Cdc14, Drosophila, Sensilla, Sperm, Chemosensation, Mechanosensation

## Abstract

Cdc14 is an evolutionarily conserved serine/threonine phosphatase. Originally identified in *Saccharomyces cerevisiae* as a cell cycle regulator, its role in other eukaryotic organisms remains unclear. In *Drosophila melanogaster*, Cdc14 is encoded by a single gene, thus facilitating its study. We found that Cdc14 expression is highest in the testis of adult flies and that *cdc14* null flies are viable. *cdc14* null female and male flies do not display altered fertility. *cdc14* null males, however, exhibit decreased sperm competitiveness. Previous studies have shown that Cdc14 plays a role in ciliogenesis during zebrafish development. In Drosophila, sensory neurons are ciliated. We found that the Drosophila *cdc14* null mutants have defects in chemosensation and mechanosensation as indicated by decreased avoidance of repellant substances and decreased response to touch. In addition, we show that *cdc14* null mutants have defects in lipid metabolism and resistance to starvation. These studies highlight the diversity of Cdc14 function in eukaryotes despite its structural conservation.

## Abstract (precis)

**Summary:** The Cdc14 phosphatase has been implicated in cell cycle regulation in *S. cerevisiae*. We show that Drosophila *cdc14* mutants are viable, but exhibit defects in sperm competition, chemosensation, and mechanosensation.

## INTRODUCTION

Cdc14 phosphatases are a well conserved family of proline-directed serine/threonine phosphatases (Mocciaro et al., 2010). Initially identified in *Saccharomyces cerevisiae* as an essential cell cycle protein (Stegmeier and Amon, 2004), Cdc14 functions to antagonize cyclin-dependent kinase (CDK)-mediated phosphorylation events (Machin et al., 2016; Mocciaro et al., 2010; Queralt and Uhlmann, 2008; Stegmeier and Amon, 2004). Despite its conservation, Cdc14 orthologs are not essential for cell division in all organisms, although they play important roles in an array of biological processes, including chromosome segregation (Clemente-Blanco et al., 2011; Machin et al., 2016; Mocciaro et al., 2010; Stegmeier and Amon, 2004), cytokinesis (Clifford et al., 2008), centrosome duplication (Mocciaro et al., 2010; Rüthnick and Schiebel, 2016), mitotic exit (Wolfe and Gould, 2004), transcription (Clemente-Blanco et al., 2009, 2011; Guillamot et al., 2011; Papadopoulou et al., 2010), the DNA damage response (Mocciaro et al., 2010), and ciliogenesis (Clément et al., 2011, 2012). Although they have been much studied, a comprehensive understanding of Cdc14 phosphatases in higher eukaryotes in particular is still lacking.

A thorough dissection of the role(s) of Cdc14 phosphatases in metazoans is complicated by the existence of multiple Cdc14 paralogs in vertebrates (Table S1) (Clément et al., 2011; Kaiser et al., 2004; Krasinska et al., 2007; Li et al., 2000; Mocciaro et al., 2010). For example, human Cdc14 phosphatases are encoded by three different genes, *CDC14A*, *CDC14B*, and *CDC14C* (Clément et al., 2011; Kaiser et al., 2004; Krasinska et al., 2007; Li et al., 2000; Mocciaro et al., 2010). Knockout studies of individual human CDC14 genes failed to demonstrate growth or mitotic defects, possibly reflecting functional redundancy between the paralogs (Berdougo et al., 2008; Mocciaro et al., 2010). However, it is still unclear whether the cellular functions of Cdc14 paralogs are fully redundant or simply overlapping. It is clear that they have distinct intracellular locations with CDC14A at centrosomes and CDC14B in the nucleolus of interphase cells (Clément et al., 2011; Kaiser et al., 2004; Krasinska et al., 2007; Li et al., 2000; Mocciaro et al., 2010), and they have been assigned some distinct functions. While CDC14A has been implicated in cytokinesis, transcriptional repression, and DNA damage repair, CDC14B is implicated in G1-phase length, centriole duplication, spindle stability, zygotic genome activation, DNA damage repair and checkpoint response (Buffone et al., 2014; Cho et al., 2005; Clemente-Blanco et al., 2011; Rodier et al., 2008; Wu et al., 2008). The biological role of CDC14C is currently unknown (Rosso et al., 2008).

Like yeast, the roundworm, *Caenorhabditis elegans*, has only one identified Cdc14 orthologue. The lack of multiple paralogs makes the roundworm an attractive organism to gain a comprehensive understanding of Cdc14 phosphatase function in a higher eukaryote. However, in *C. elegans*, Cdc14 functions in a manner unrelated to that in any other organism reported to date – to promote cellular quiescence of specific precursor cells (Cueille et al., 2001; Saito et al., 2004).

The common fruit fly, *Drosophila melanogaster* also has a single gene that encodes Cdc14 (*Dmel\cdc14*), the role of which has not yet been reported (Fisher et al., 2012). Herein, we demonstrate that the Drosophila *cdc14* gene plays a role in sperm competitiveness, chemosensory reception, mechanosensory reception, fat body metabolism, and longevity during starvation conditions. This array of phenotypes associated with loss of *cdc14* function is once again distinct from those identified in any other organism, thereby highlighting a remarkable functional versatility for such a conserved protein.

## RESULTS

### *cdc14* expression is highest in the testis

**Fig. 1.**
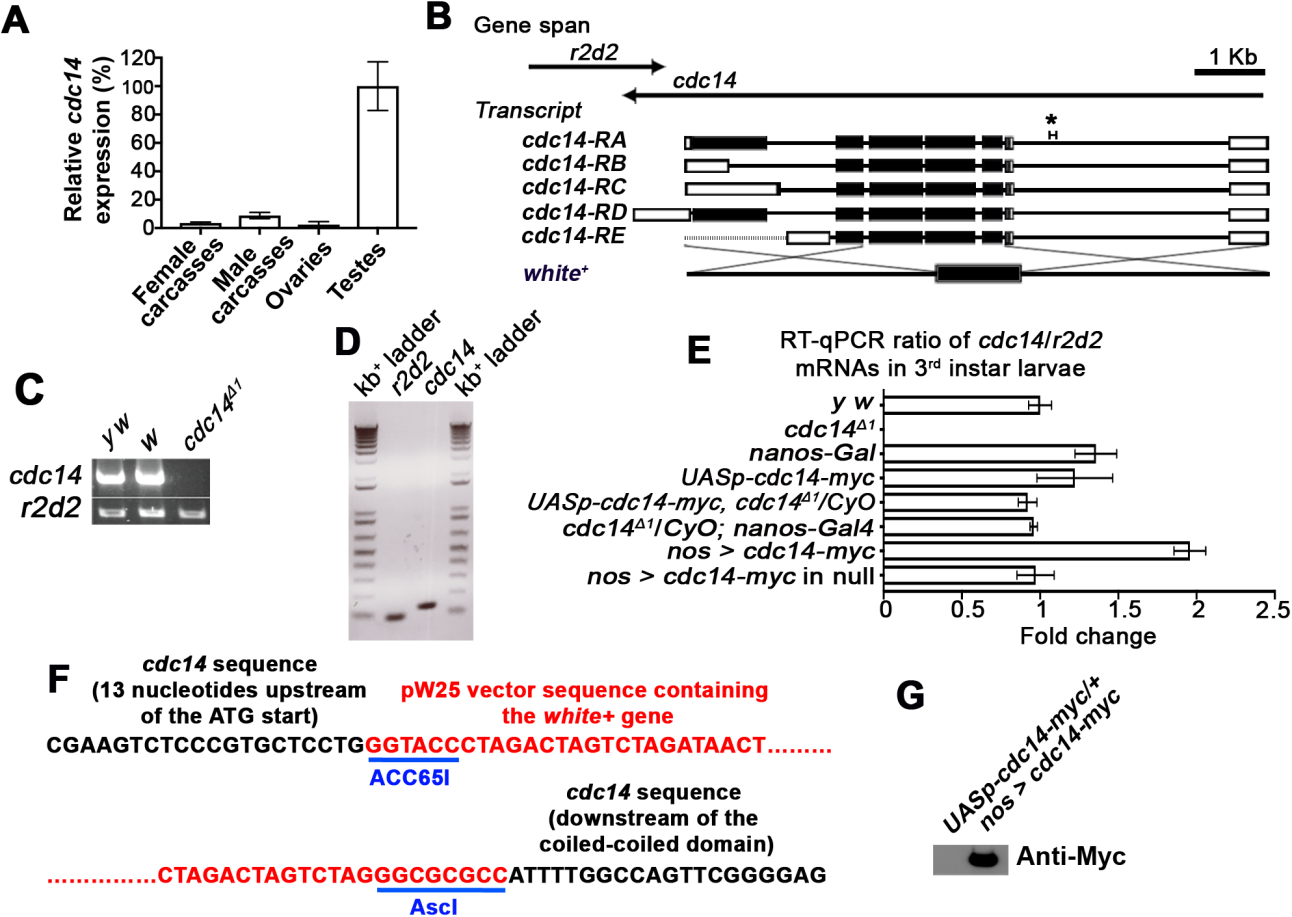
**High expression of *cdc14* in the testes and generation of Drosophila *cdc14* null mutants by homologous recombination.** (A) Relative expression of endogenous *cdc14* in adult flies as determined by RT-PCR. The highest level of expression is in the testes. *cdc14* expression was normalized to *Rp49*. cDNA was generated from adult carcasses (*n*≥50) or excised gonads (*n*≥200, *n*=3 independent biological replicates with *n*=3 technical replicates). (B) Structure of the Drosophila *cdc14* and its five alternative transcripts. Black boxes are exons, white boxes are UTRs, and lines are introns. Ends-out homologous recombination of *cdc14* was used to replace *cdc14* with the *white^+^* gene. Asterisk (*) indicates the 371 bp (2L:7,807,273 to 7,87,543) region of *cdc14* used for PCR depicted in C. A region (2 kb; not shown) of the overlapping housekeeping gene, *r2d2*, was used as a positive PCR control. Control *r2d2* PCR product is 2 kb. *r2d2* is upstream of *cdc14* (PCR region not depicted). *White^+^* gene is not to scale. (C) A *cdc14* null line was generated in a *y w* background and verified by PCR amplification of genomic DNA. The control lines, *y w* and *w^1118^*, and the adjacent housekeeping gene, *r2d2*, were used as positive controls. The *cdc14^Δ1^* null allele was used for all subsequent experiments and for generation of the rescue line. The gel is a representative result from *n*=3 replicates. (D) Final products from the RT-qPCR reaction of *y w* third instar larvae run on a 1% agarose DNA gel. Only a single product was amplified, suggesting high specificity of the primers used in E. (E) Fold changes of *cdc14* mRNA normalized to *r2d2* mRNA. The level of expression is normalized to the *y w* control. No *cdc14* expression was detectable in the *cdc14 null* line, but *r2d2* expression was equivalent to that of the *y w* control line. cDNA was generated from late third instar larvae (*n*≥30, *n*=3 independent biological replicates with *n*=3 technical replicates). (F) Nucleotide sequence of the boundaries of the *cdc14* null mutation. Two of the restriction endonuclease sites (ACC65I and AscI) used for cloning the two homologous arms of *cdc14* into the pW25 vector for recombination are shown. (G) Anti-Myc immunoblot of 0–2 h old embryos demonstrates expression of *UASp-cdc14-myc* using the *nanos-Gal4* driver.

Tissue expression data from the FlyAtlas indicate differential expression of *cdc14* with a sevenfold higher level of *cdc14* mRNA in the testis compared to the next highest expressing organ, the brain. To verify organ-specific levels of expression, we performed quantitative-PCR of whole adult carcasses, ovaries, and testes. Consistent with FlyAtlas, we found testis-specific expression of *cdc14* to be approximately eleven times higher than whole male carcass expression (Fig. 1A).

### Generation of *cdc14* knockout lines in *D. melanogaster*

Homologous recombination was used to generate a *cdc14* null allele, designated *cdc14^Δ1^*, in a *y w* line by replacing a portion of the *cdc14* gene (spanning from the 5′-UTR to just downstream of exon 6) with the *white^+^* gene (Fig. 1B). We then generated a stable homozygous knockout line (hereafter referred to as *cdc14* null) with confirmation of knockout by genomic PCR (Fig. 1C). The housekeeping gene, *r2d2*, adjacent to *cdc14* served as a control (Fig. 1C). Additionally, while *cdc14* mRNA was detected in wild-type flies by quantitative PCR (Fig. 1D,E), there was no detectable *cdc14* mRNA in the *cdc14* null flies (Fig. 1E). Further confirmation of the knockout was performed by sequencing the boundaries of the *cdc14* gene mutation (Fig. 1F).

### Knockout of *cdc14* is well tolerated

*cdc14* null flies are viable and reach adulthood. Female *cdc14* nulls crossed to male *cdc14* nulls lay eggs at the same rate as the *y w* control line, regardless of the age of the females (Fig. S1A,B). The average number of eggs produced per laying female is not significantly different (Fig. S1A,C). The progeny of female *cdc14* nulls crossed to male *cdc14* nulls are viable. An equivalent proportion of eggs developed into 3-day-old adults when compared to the control line (Fig. S1D,E,F,G). Finally, no difference in the ratio of male to female progeny was observed (Fig. S2C). Our data suggests that Cdc14 is not an essential gene in Drosophila.

### Overexpression of Cdc14-Myc

To determine whether overexpression of Cdc14 could provide insight into its function, we generated an inducible cMyc-tagged Cdc14 expression line (*UASp-cdc14-myc*) and verified expression of Cdc14-Myc by immunoblot analysis; this transgenic line was used for overexpression and rescue experiments (Fig. 1G; Fig. S2A,B). Nanos is active in germline formation, oocyte maturation, and early embryogenesis (Forbes and Lehmann, 1998; Kobayashi et al., 1996; Wang and Lehmann, 1991), making a *nanos* (*nos*) driver appropriate to rescue *cdc14* loss, especially in the germ line. We found that introduction of *nos>cdc14-myc* in a *y w* background resulted in a twofold increase in *cdc14* mRNA levels compared to the *y w* control line at the late third instar stage of larval development and very closely approximated wild-type levels of expression in a *cdc14* null background (Fig. 1E). Immunoblotting confirmed expression of Cdc14-Myc in *nos>cdc14-myc* in third instar larvae (Fig. S2A,B). All rescue experiments described herein were performed using the *cdc14* null line with *nos>cdc14-myc* expression (‘rescue line’).

### *cdc14* knockdown does not affect the ratio of male-to-female progeny

The relatively high expression of *cdc14* in the Drosophila testis compared with other tissues raised the possibility that knockout or overexpression may specifically affect the health of males. However, no significant differences between the ratios of male-to-female offspring were observed (Fig. S2C,D). This finding indicates that neither the insertion/expression of *UASp*-*cdc14-myc* nor the loss of *cdc14* affect the viability of male offspring.

### Cdc14 is not required for Drosophila spermatogenesis

To test the fertility of *cdc14* null flies, we crossed male *cdc14* nulls to control females and assessed the number of offspring. We observed no significant differences in the number of progeny produced by *cdc14* null versus control males at either 0–5 or 6–11 days of age (Fig. S3A). These data suggest that *cdc14* loss does not affect male fertility.

Given that *cdc14* expression is highest in Drosophila testes relative to other tissues and that Cdc14A and Cdc14B are involved in ciliogenesis in zebrafish (Clément et al., 2011, 2012), we tested whether Cdc14 is involved in Drosophila spermatogenesis. Primary cilia first form in apolar spermatocytes and persist through the early spermatid stage, disassembling only partially at the end of spermatogenesis when the centriole at the cilium forms the base of the flagella axoneme (Riparbelli et al., 2012). These primary cilia are therefore thought to be precursors of the spermatid flagellum, although their role in spermatogenesis is unclear (Riparbelli et al., 2012). The majority of cells in the testis are ciliated; however, the function of the cilia during the meiotic prophase is unknown.

We initially examined the testis as a whole for potential gross morphological defects. Examination of bright-field images revealed no observable morphological differences between *cdc14* null and control testes (Fig. S3B). Immunostaining of testes for alpha-tubulin showed no notable differences in sperm morphology or number during spermatogenesis (Fig. S3C). Additionally, using *nos>cdc14-myc* animals, we observed localization of Cdc14-Myc protein primarily to the head of mature sperm bundles in the testes (Fig. S3D). Analysis of spermatids and mature sperm revealed no observable differences between *cdc14* nulls and controls (Fig. S3E,F). Thus, we conclude that *cdc14* is not essential for formation of mature sperm in Drosophila.

### Loss of *cdc14* does not affect Drosophila oogenesis or embryogenesis

Previous studies indicated that Drosophila *cdc14* is maternally contributed with diffuse localization in early larvae (Fisher et al., 2012; Keil, 1997). We performed immunolocalization studies using the *UASp-cdc14-myc* transgenic line under the control of a maternal *nos-Gal4* driver. Consistent with the *in situ* studies, we found that the cMyc-tagged Cdc14 protein is diffusely localized around mitotic nuclei in the early embryo (Fig. S4A).

We tested the effects of Cdc14 on viability and early development. We found that male and female *cdc14* null flies are fertile and the offspring of *cdc14* null parents are viable (Figs S1B,G and S3A). We found no significant effects of Cdc14 loss on the embryo's aspect ratio (length:width) (Fig. S4B). Thus, we conclude that Cdc14 does not play an essential role in early oogenesis or embryogenesis of Drosophila.

### Cdc14 is not required for cell cycle checkpoint activation or DNA damage repair

Cdc14 phosphatases are reported to play a role in DNA damage repair and the checkpoint response in human cells (Mocciaro et al., 2010). We observed no increase in single or double strand DNA breaks in response to ionizing radiation as determined by the amount of TUNEL staining in wing discs from *cdc14* null third instar null larvae (Fig. S4C). To determine whether Drosophila Cdc14 is involved in DNA checkpoint regulation, we assessed the mitotic index of eye discs from irradiated larvae of *cdc14* null animals by immunostaining for the mitosis specific marker phospho-histone H3 (pH3). Eye discs from *cdc14* null flies showed a reduction in pH3 similar to that of control flies following irradiation (Fig. S4D), suggesting normal activation of a DNA damage checkpoint. As positive control, discs from homozygous animals that are null for an allele of the checkpoint gene, *mei-41^RT^*, showed no reduction in proliferation. These results suggest that *cdc14* is not required for mitotic checkpoint activation or DNA damage repair in Drosophila.

### Sperm competiveness is decreased in *cdc14* null flies

The fertility experiments performed above reflect reproduction in an optimized and controlled laboratory environment. In the wild, Drosophila males compete with each other by promoting removal or inactivation of the previous male's sperm (Price et al., 1999). Therefore, we performed a sperm competition assay to assess ‘sperm fitness’. By sequentially mating a white-eyed male and red-eyed male to a white-eyed female, competitiveness was determined by counting the number of red-eyed (heterozygous for red pigment genes) or white-eyed offspring. The *cdc14* null, rescue, and overexpression lines are all homozygous for a gene that produces red pigment in the eye. The *y w* line has white eyes, as it is homozygous for a loss of function allele of the *white* gene and does not produce red pigment.

**Fig. 2.**
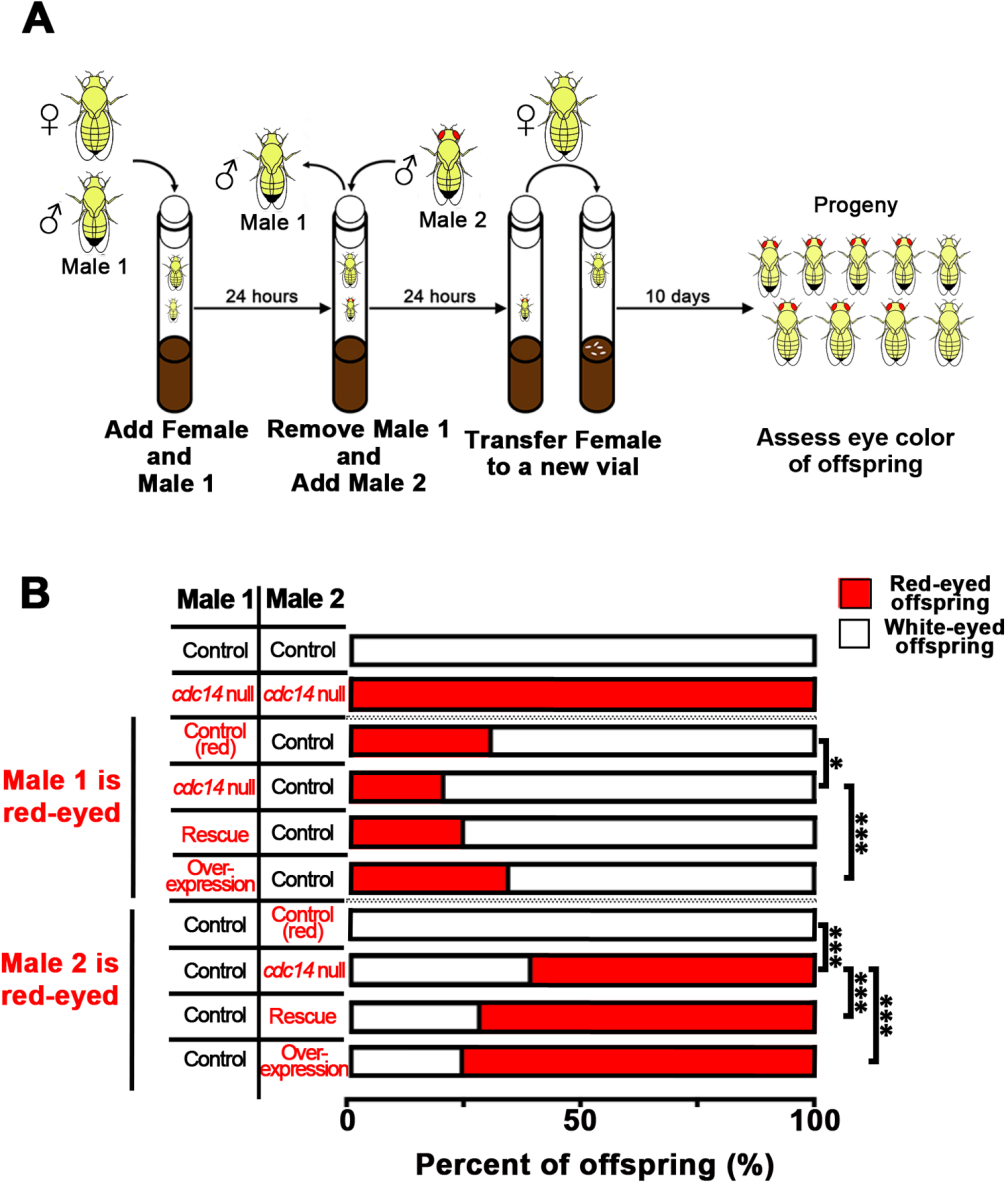
***cdc14* null males exhibit decreased sperm competition.** (A) An example of a sperm competition assay in which a single *y w* virgin female (white-eyed) is mated to a single male (white-eyed) for 24 h. The male is then removed and the female is mated to a second male with red-eyes for 24 h. The female is then transferred to a fresh vial and allowed to lay eggs. The female is removed, and offspring are allowed to develop and assessed for eye color (red or white). The assay is repeated using a red-eyed male first and then a white-eyed male. (B) A control experiment was performed using white-eyed *y w* males for both the first and second males. A second control experiment was performed using red-eyed *cdc14* null males for both the first and second males. The *cdc14* null males are less competitive compared to control males regardless of whether they are the first or second male to mate. Results for a single representative replicates (*n*≥15 vials per cross) are shown. Additional data can be found in Fig. S5B. Data were analyzed using a Chi-squared test with Bonferroni correction. Six pairwise comparisons were made. Red-eyed control males were compared to the *cdc14* null, rescue, or overexpression males; *cdc14* null males were compared to rescue or overexpression male; and rescue males were compared to overexpression males. *P<0.009 and ***P<0.0002.

When mated to a white-eyed female, the percentage of offspring produced by the *cdc14* null was significantly less than that of the control groups, regardless of whether the *cdc14* null male was the first or second male to mate (Fig. 2; Fig. S5B). This result suggests that, in spite of the lack of notable morphological differences between *cdc14* null and control sperm, *cdc14* null sperm are less efficient than wild-type sperm in conferring reproductive competitiveness.

It is possible that seminal fluid proteins could induce changes in female behavior and physiology, such as sexual receptivity, ovulation, and egg-laying rates, and these changes could confound a sperm competition assay (Avila et al., 2011). We found that the cdc14 stock had a normal egg-laying rate (Fig. S1B,C,E,F). The proportion of *cdc14* null males that mated at least once in the sperm competition assay was comparable to controls (Fig. S5A). However, we cannot rule out that *cdc14* nulls mated fewer times than controls within the 24 h period.

The seminal fluid protein, sex peptide (SP), is transferred with the sperm to the female reproductive tract, where it is bound to the tail of the sperm and plays a role in sperm storage (Avila et al., 2010; Avila and Wolfner, 2009; Peng et al., 2005; Rogers et al., 2009). SP null males have been reported to give rise to more progeny compared to controls when mated first in specific competition assays (Avila et al., 2010). In contrast, we found that *cdc14* null males gave rise to fewer progeny compared to controls when they were mated first (Fig. 2; Fig. S5B). Thus, it is possible that Cdc14 may function to inhibit SP release or the association of SP with the ciliary tail of the sperm.

### Loss of *cdc14* does not affect Drosophila path-length, coordination, or locomotion

The decrease in sperm competitiveness may be the result of coordination or locomotion defects inhibiting mating behaviors. Drosophila are negatively geotactic, and the adult climbing assay is a motor assay that takes coordination into account (Ali et al., 2011; Nichols, 2015; Nichols et al., 2012). When coordination is disrupted, flies should climb slower and/or fewer flies should rapidly climb the chamber walls (Nichols, 2015). We found that the climbing behavior of *cdc14* nulls was not significantly different than that of control *y w* control (Fig. S6A). Furthermore, path-length (distance traveled by a larva on yeast within a defined period) and the number of body wall contractions, both measures of locomotion (Anreiter et al., 2016; Nichols et al., 2012), did not significantly differ between *cdc14* null and control larvae (Fig. S6B,C). These results suggest that Cdc14 does not play a role in path-length, coordination, or locomotion in Drosophila.

### Drosophila *cdc14* null larvae have decreased mechanosensation

In Drosophila*,* spermatozoa and sensory neurons are the only ciliated cells (Ma and Jarman, 2011). Type I sensory neurons (sensilla) have modified cilia that act as a site for sensory reception and transduction (Field and Matheson, 1998; Keil, 1997; Laurençon et al., 2007; Lee et al., 2008; Ma and Jarman, 2011; Riparbelli et al., 2012). In accordance with a role in zebrafish ciliogenesis (Clément et al., 2011, 2012), we tested whether *cdc14* null sensory neurons have decreased function compared to that of control flies. Mechanosensation and chemosensation are the two primary functions of Type I sensory neurons (Brody, 1999). To test the functionality of the mechanosensory neurons, we used a touch insensitivity assay in third instar larvae (Fig. S7). Control larvae demonstrated a normal distribution of touch response scores (Fig. S8A). In contrast, *cdc14* larvae showed a broad distribution of touch response scores and a decreased average score, suggesting a distinct loss of touch sensitivity (Fig. S8A,B). The loss of touch sensitivity in *cdc14* null larvae was partially rescued by maternally contributed *cdc14* (*cdc14^Δ1-Maternal^*; Fig. S8B). These data suggest a role for *cdc14* in the function and/or formation of ciliated Type I sensory neurons controlling mechanosensation.

### Gustation in Drosophila larvae is modulated by *cdc14*

Drosophila gustatory neurons are also Type I sensory neurons. Drosophila larvae have more than 81 external chemosensory gustatory neurons (including 37 neurons in the head) (Stewart et al., 2015). In order to test the functionality of the chemosensory neurons, we assessed the ability of larvae to detect and avoid the bitter substance, quinine (Wu et al., 2005). Larvae were placed for 5 min on a nutritious yeast paste containing food coloring, and the number of larvae with dyed food in >50% of their intestines were scored as feeding (Fig. S9A). In the absence of quinine, three times fewer *cdc14* null larvae had fed compared to control larvae (Fig. S9A). In the presence of quinine, only 5% of control larvae had fed, whereas the percentage of fed *cdc14* null larvae remained unchanged at 15–17% (Fig. S9A). These findings indicate that loss of cdc14 results in both a loss of chemosensation and reduced but indiscriminate feeding.

A second test was used to verify the changes in gustatory sensation and to test the ability of the animals to discriminate between attractive (sucrose) and repellant (quinine) substances (Fig. S9B,C,D). In the absence or presence of sucrose across an entire plate, *cdc14* null larvae demonstrated a significant decrease in aversion to quinine when compared to controls (Fig. S9B,D). Furthermore, this discrimination against quinine returned to nearly wild-type levels in *cdc14^Δ1-Maternal^* larvae. When given the choice between sugar and quinine, no statistically significant differences were found between the *cdc14* null larvae and controls (Fig. S9C). No differences between the sexes were observed under these testing conditions.

In contrast to larvae, no difference in quinine avoidance was observed between *cdc14* null and control adults (Fig. S10A). Our results suggest that *cdc14* null larvae have the capacity to identify certain attractive substances (sucrose), but not repellant substances (quinine), possibly due to a defect in the functionality of the gustatory sensilla.

### Photoreception is unaffected in the *cdc14* null larvae

To test if the mechanosensory and chemosensory behaviors in *cdc14* null larvae were specific to ciliary function (rather than non-ciliary-mediated functions such as mobility), we tested photoreception, which is not mediated by ciliated neurons. Drosophila are negatively phototactic (Lilly and Carlson, 1990). Late third instar larvae were exposed to light with a choice between opaque-black and clear agarose backgrounds. We observed no differences between the *cdc14* null, control, and rescue larvae (Fig. S10B). These results suggest the observed defects in *cdc14* null animals are specific to ciliated cell types (e.g. Type I sensory neurons and sperm).

### *cdc14* confers resistance to starvation and modulation of lipid metabolism

In a Drosophila genome-wide RNAi screen for genes involved in adiposity, *cdc14* was previously identified as a potential regulator of triglyceride metabolism (Pospisilik et al., 2010). Drosophila store energy in the form of triglycerides and glycogen; growth, reproduction, and normal energy expenditure under extended non-feeding periods (e.g. starvation) are dependent upon proper lipid metabolism (Arrese and Soulages, 2010). Alterations in fat metabolism manifest as changes in the size of the fat body or the abundance of lipid droplets (Figueroa-Clarevega and Bilder, 2015).

We dissected and analyzed the larval fat body, the major triglyceride storage organ (Figueroa-Clarevega and Bilder, 2015; Gutierrez et al., 2007). Staining with Oil Red O revealed large and abnormally shaped fat bodies in the *cdc14* null larvae in contrast to the controls (Fig. S11A,B). Staining with Nile Red showed a similar effect (Fig. S11C).

Previous studies have demonstrated that female flies are more resistant to starvation and have a higher lipid content than males (Huey et al., 2004; Jang et al., 2014). Thus, defects in triglyceride regulation may affect the resistance of the animals to nutrient deprivation. We found that control females have a slightly longer lifespan (>4.25 h) compared to *cdc14* null females under starvation conditions (Fig. S11D). The decrease in lifespan under starvation conditions and alterations in lipid droplet morphology observed in *cdc14 null* flies suggest that Cdc14 may play a role in the regulation of metabolism in Drosophila.

## DISCUSSION

In the current study, we used ends-out homologous recombination to knock out the single Drosophila *cdc14* gene (Fisher et al., 2012; Gong and Golic, 2003). We have found that Drosophila Cdc14 confers several competitive advantages. Despite the fact that *cdc14* null males are fertile, they have a decreased capacity to compete with other males for mating. Loss of larval gustatory chemosensation and mechanosensation results in reduced and indiscriminate feeding behaviors, presumably leading to inadvertent feeding on a toxic food source. Our findings suggest that in times of food scarcity, *cdc14* nulls have a shorter lifespan than wild-type flies, possibly due to altered lipid metabolism and/or feeding behaviors. This constellation of defects associated with loss of *cdc14* in Drosophila may provide an explanation for its conservation in metazoans.

To our knowledge, the present study is the first characterization of a Drosophila *cdc14* null mutation. Similar to *S. pombe* and *C. elegans*, *D. melanogaster cdc14* is not an essential gene. Flies lacking *cdc14* are viable, and they do not display phenotypes that have been observed in other organisms (Mocciaro and Schiebel, 2010). For example, we were unable to demonstrate that *cdc14* participates in the DNA damage repair response, and loss of *cdc14* did not alter growth or lead to any obvious perturbations in mitosis or more generally in cell proliferation. Specifically, we did not observe any centrosomal or mitotic spindle defects or any defects in chromosome segregation or cytokinesis (data not shown).

We did not detect morphological differences in sperm formation between *cdc14* null and wild-type males. However, we show that sperm competiveness is decreased in *cdc14* null flies, suggesting an effect on sperm ciliary function (Brody, 1999). These findings merit further detailed molecular study of the role of *cdc14* in sperm ciliary function.

## MATERIALS AND METHODS

### Generation of *cdc14* null by homologous recombination

For recombination, the homologous arms for *cdc14* were cloned into the pW25 vector. The left homologous arm was generated using the forward primer CG7134-NotI (5′-AGCAGCGGCCGCTACATCGCGGTTCGTGTCACCG-3′ and the reverse primer CG7134-ACC65I 5′-TACCGGTACCCAGGAGCACGGGAGACTTCGAC-3′). The right homologous arm was generated using the forward primer CG7134-AscI (5′-AGCAggcgcgccATTTTGGCCAGTTCGGGGAGCAG-3′) and the reverse primer CG7134-BsiWI (5′-TACCCGTACGTCTCCACCAATTTGTAGGTGGG-3′). The construct covers a 9035-bp region of chromosome 2L where the left arm ends 13 bp upstream of the ATG and the right arm starts immediately after the coiled-coil domain encoded by *cdc14*. This construct was used to excise the 5′-UTR and exons 1 through 6 of *cdc14*-RA and *cdc14*-RD and the 5′-UTR, exons 1 through 5, and 3′-UTR of *cdc14*-RB, *cdc14*-RC, and *cdc14*-RE (2L:7802415 to 7810697) and replace it with the *white^+^* cDNA via ends-out-homologous recombination (Gong and Golic, 2003; Maggert et al., 2008).

Knockout lines were verified by PCR of *r2d2* and *cdc14* using the forward 5′-TTGATAGAGCGCTCTCTCGT-3′ and reverse 5′-CGGATGGATGGAAGTATGTA-3′ primers for *r2d2* (Liu et al., 2006) and the forward 5′-CATCGCTGTATTTCCACCCAC-3′ and reverse 5′-AAGGCATCACTCGCGATCC-3′ primers for *cdc14*.

For PCR and sequencing of the boundaries of the *cdc14* mutation, the forward 5′-CGAAGTCTCCCGTGCTCCTG-3′ and reverse 5′-CGACGAAGCGCCTCTATTTA-3′ primers were used for the left recombination boundary and the forward 5′-TCCGGTTGTTTTCGTGCTCA-3′ and reverse 5′-CTCCCCGAACTGGCCAAAAT-3′ primers were used for the right recombination boundary.

### DNA constructs

cDNA clone GH01148 encoding *cdc14*-B was obtained from the Drosophila Gene Collection (Stapleton et al., 2002). *UASp-cdc14-myc* was created by subcloning the amplified coding sequence from GH01148 into a modified version of UASp that confers a C-terminal Myc tag (Rørth, 1998).

### Drosophila stocks

Stocks were maintained at 25°C using standard techniques (Greenspan, 2004). The *y^1^ w^1118^*, *w^1118^*, *mei-41^RT1^* (FlyBase ID: FBal0046106), and red-eyed control [*y w*; *FLAG-Mcm4* (*BAC#1 attP40*): from the Nordman lab, Vanderbilt University] lines were used as controls. The *UASp-cdc14-myc* transgenic line was generated by *P*-element mediated insertion via embryo injection in the *y w* background using standard methods (Rubin and Spradling, 1982). Overexpression of *UASp-cdc14-myc* was driven by crossing with *nanos-Gal4* (FlyBase ID: FBst0032563) or *tubP-Gal4* (FlyBase ID: FBtp0002651) flies. The *UASp-cdc14-myc, cdc14^Δ1^* line was established by recombination using standard methods. The *cdc14^Δ1^*; *nanos-gal4* line was established by performing standard genetic crosses. *cdc14^Δ1^* null larvae with maternally contributed *cdc14* (*cdc14^Δ1-Maternal^*) were obtained by crossing *cdc14^Δ1^*/*CyO* females to *cdc14^Δ1^* homozygous males. All transgenic lines were isogenic.

The *cdc14* gene is located on chromosome 2L:7,801,668 to 7,810,703 (FlyBase ID: FBgn0031952, FlyBase build: FB2018_02) and has five alternative transcripts, the longest of which encodes a 1052 amino acid protein (Fig. 1B) (Gramates et al., 2017). The *cdc14* gene is flanked by the housekeeping gene *r2d2* (2L:7,800,147 to 7,802,098).

### Quantitative PCR

cDNA was generated from adult carcasses, late third instar larvae, and excised gonads. Samples were homogenized in 1 ml RNA Stat-60 and cleaned up by chloroform extraction. cDNA was prepared using the High Capacity cDNA Reverse Transcription Kit (Applied Biosystems, Foster City, USA) with the supplied random primers. qPCR was performed using the GoTaq^®^ qPCR Master Mix (Promega, Madison, USA) on a CFX96 qPCR machine from Bio-Rad. cDNA was prepared in triplicate using samples from separate crosses, and qPCR was performed using three technical replicates.

The forward 5′-GAGATGCAGGAAGACCGATTAT-3′ and reverse 5′-CTCATCGACGCTGAAGTAGTG-3′ primers were used to assess relative *cdc14* mRNA levels, which were normalized to Dmel\*Rp49* expression levels using the 5′-GAGATAGAGGCCCTTGGAAATG-3′ forward and 5′-CAGATCACCCACAGTCGAATC-3′ reverse primer.

### Immunoblotting

Embryo lysates for immunoblotting were generated as previously described (Hainline et al., 2014). For late third instar larvae, pools of ten were collected and homogenized in 200 μl of running buffer (Fig. 1F, Fig. S2B,C), and protein equivalent to one-half of a larva (10 μl) was used. All samples were run on ExpressPlus™ PAGE gels 4–20% from GenScript. Membranes were probed with mouse anti-Myc (9E10, 1:100; Santa Cruz Biotechnology, catalog #sc-40) and mouse anti-alpha tubulin (DM1α, 1:2500; Sigma-Aldrich, catalog #T6199) primary antibodies and anti-mouse horseradish peroxidase (1:5000) secondary antibody followed by visualization of signal on a LI-COR C-DiGit^®^ Blot Scanner using SuperSignal™ West Femto Maximum Sensitivity Substrate or HyGLO™ Quick Spray.

### Assessment of female fertility

Males were allowed to mate with virgin females for 3 or 7 days within chambers on grape juice plates (3% agar, 25% grape juice, 0.3% sucrose). Individual females were then transferred to a standard culture vial and allowed to lay eggs for 24 h followed by assessment of the number of eggs per vial. The average number of eggs per female was determined by counting the total number of eggs collected and dividing by the number of females that laid eggs.

### Progeny survival assay

Males were allowed to mate with virgin females for 3 days within chambers on grape juice plates (3% agar, 25% grape juice, 0.3% sucrose). Groups of ten females were then transferred to a single standard culture vial and allowed to lay eggs for 24 h followed by the removal of adults and determination of the number of eggs collected per vial. Eggs were allowed to develop through larval stages to adulthood, and the number of adults alive at 3 days post-eclosion was assessed. The percentage of eggs that survived to 3-day-old adults was determined by dividing the number of 3-day-old adults by the number of eggs laid.

### Assessment of progeny sex

Mendelian inheritance assays were performed by mating equal numbers of 3-day-old males and virgin females under standard conditions. Parental flies were removed 9 days after setting up the cross. Adult progeny were collected daily and counted from day 10 to day 20.

### Male fertility assay

Fertility assays were performed by mating equal numbers of 0–5- or 6–11-day-old males (control or *cdc14* nulls) with control virgin females under standard conditions. Parental flies were removed 9 days after setting up the cross. Adult progeny were collected and counted daily from day 10 to day 20.

### Sperm competition assay

Sperm competition was performed based on a modification of a previously described assay (Yeh et al., 2013). Virgin males and females were collected and aged for 3 days on standard food supplemented with extra yeast paste. Single mating pairs were transferred to new vials and allowed to mate for 24 h. The first male was then removed, and a second 3-day-old virgin male was added to the vial and allowed to mate for 24 h. The female was then transferred to a fresh standard culture vial and allowed to lay eggs at 25°C for 9 days, at which point the female was removed. Eclosed adult progeny were collected and counted daily over the next 10 days and eye color was assessed. The experiments were blinded to the genotypes of the red-eyed males used in these experiments.

### Assessment of spermatids and mature sperm

Testes were isolated from 1–3-day-old males as previously described (Zamore and Ma, 2011). Testes were snipped and squashed as previously described (Sitaram et al., 2014). Spermatids and mature sperm were observed using bright field microscopy.

### Immunostaining

Testes were collected from 1–3-day-old males as previously described (Zamore and Ma, 2011). Testes were snipped at level 2 and immunostained as previously described (Sitaram et al., 2014). Anti-Myc (9E10, 1:100; Santa Cruz Biotechnology, catalog #sc-40) and anti-tubulin (DM1α, 1:200; Sigma-Aldrich, catalog #T6199) primary antibodies were used in combination with goat anti-mouse Cy2 secondary (1:400; Invitrogen, catalog #A-11004).

Localization of Myc-tagged Cdc14 protein in 0–2 embryos laid by females carrying the *UASp-cdc14-myc* and *nanos-Gal4* transgenes was assessed by immunofluorescence using standard conditions. Anti-Myc (9E10, 1:100; Santa Cruz Biotechnology, catalog #sc-40) and rat anti-alpha tubulin (1:200; Accurate Chemical & Scientific, Westbury, USA, catalog #MCA77G) primary antibodies were used in combination with goat anti-mouse Cy2 (1:400; Invitrogen, catalog #A-11004) and goat anti-rat Cy3 (1:400; Abcam, catalog #ab6953) secondary antibodies.

### Microscopy

Bright field images were obtained using a Stemi 2000-CS microscope (Zeiss, Oberkochen, Germany) with an Olympus DP72 camera. Fluorescent images were obtained using a Nikon Eclipse 80*i* microscope with a Cool SNAP ES camera (Photometrics, Tucson, USA). Images were analyzed in Fiji or Photoshop.

### Egg aspect ratio

Adults were placed in egg-laying chambers over a grape juice plate (3% agar, 25% grape juice, 0.3% sucrose) and allowed to lay for 1 h. Plates were collected, imaged, and assessed by a blinded experimenter. The measuring tool in Fiji was used to determine the ratio of the anteroposterior to sagittal axes of imaged 0–1 h embryos.

### Larval path-length

Analysis of larval path-length was based on a modification of a previously described assay (Anreiter et al., 2016). Early third instar larvae were washed in PBS and placed on a 1% agarose plate. Larvae were allowed to move freely for 1 min. Plates were imaged, and the length of the path taken was assessed in Fiji as previously described (Anreiter et al., 2016).

### Body wall contraction assay

Determination of body wall contraction was based on a modification of a previously described assay (Nichols et al., 2012). Early third instar larvae were washed in PBS and placed on a 1% agarose plate. Larvae were observed for 1 min under a dissection microscope, and the number of peristalsis contractions was scored. A single contraction was defined as a full anterior to posterior movement.

### Adult climbing assay

Analysis of adult climbing was based on a modification of a previously described assay (Crowther et al., 2005). Up to 30 animals at 7–10 days post eclosion were placed at the bottom of a 3 inch vial. A second vial was placed on top of the first vial, and flies were allowed to climb for 20 s. The vials were then separated, and the number of flies in the top vial were counted. The percentage of flies climbing was determined by dividing the number of animals in the top vial by the total number of animals in both vials.

### DNA damage assays

Third instar larvae were collected, washed in PBS, and left untreated or exposed to 40 Gy of ultraviolet radiation using a UV Stratalinker 1800 from Stratagene. Larvae were incubated at room temperature and dissected for wing or eye discs as previously described (Purves and Brachmann, 2007). Discs were stained with terminal deoxynucleotidyl transferase dUTP nick end labeling (TUNEL) or anti-phospho-Histone H3 (pH3) as previously described (Brodsky et al., 2000; Sarkissian et al., 2014). TUNEL- and pH3-stained samples were assessed by a blinded experimenter using Fiji. TUNEL-stained samples were assessed by measuring the fluorescence of the entire sample. pH3-stained samples were assessed by counting the number of stained cells in each sample.

### Mechanosensation assay

Touch insensitivity of larvae was assessed using a previously described assay (see Fig. S7) (Kernan et al., 1994). A human eyelash is affixed to the end of a dowel rod with tape to make an eyelash brush. Larvae were washed with PBS, placed on a 1% agarose plate, tested four times by a blinded experimenter, and assigned a scored between 0 to 4. Scores were added together to determine the final score (0–16).

### Larval yeast feeding assay

Test plates and yeast paste were prepared using blue or red food coloring as previously described (Wu et al., 2005). Early third instar larvae were washed in copious amounts of PBS, placed on the yeast paste, and allowed to feed or roam freely for 30 min. Wu et al. (2005) found that larvae feed in a bimodal fashion, either feeding persistently with substantial coloration of the gut or roaming across the plate without feeding. Larvae were scored by a blinded experimenter as feeding (>50% of the midgut full of colored yeast paste) or non-feeding (≤10% of gut filled with colored yeast past).

### Larval quinine preference assay

The quinine chemosensory assay was performed as previously described with minor alterations (Apostolopoulou et al., 2014). Plates were prepared by filling a 60 mm petri dish with 5 ml of autoclaved 0.5% agarose (minus or plus 7.5 mM sucrose) and allowed to solidify. Agarose was then removed from one-half of the plate and replaced by 2.5 ml of media (cooled to 50°C) containing quinine (minus or plus 7.5 mM sucrose). Plates were allowed to cool to room temperature and then stored in the dark at 4°C. Early third instar larvae were collected, washed in PBS, and placed near the center of the plate. Larvae were placed perpendicular to the boundary between the quinine-containing and quinine-free halves of the plate with the anterior end of each larva facing the quinine-containing half of the plate. Larvae were left undisturbed for 5 min and then scored by a blinded experimenter for location in either the quinine-containing or quinine-free side of the plate. Preference for quinine was determined as follows as previously described (Apostolopoulou et al., 2014): Preference=[(Larvae on quinine)−(Larvae on agarose)]/Total number of larvae. Preferences can range from −1 to 1, with negative values indicating avoidance of quinine and positive values indicating no avoidance of quinine.

### Chemosensation test of adults

A modified version of the previously described two-way choice behavioral assay was used (Shim et al., 2015). Virgin 3-day-old adult flies were starved for 18 h and then placed in the dark with a 96-well plate containing 1 mM sucrose plus brilliant blue FCF dye (blue; 0.125 mg/ml) or 1 mM sucrose+0.8% quinine plus sulforhodamine B dye (red; 0.2 mg/ml) in alternating wells. Feeding was allowed to proceed for 90 min, flies frozen, and carcasses analyzed for the presence of ingested dye. The experiment was then repeated with the dyes reversed.

### Phototaxis assay

Response of larvae to photostimulation was assessed as previously described with the following modifications (Lilly and Carlson, 1990). One-half of the plate contained no added food coloring, and the other half contained 1 ml of an equal-parts mixture of red, green, and blue food coloring added to 100 ml of 0.5% agarose to produce an opaque black-colored agarose. Larvae were placed on the light box and allowed to move freely for 5 min prior to assessment of their location by a blinded experimenter.

### Fat body staining

Staining for fat bodies was performed using Oil Red O as previously described (Gutierrez et al., 2007)**.** Nile Red experiments were performed using the same protocol except for the dye substitution. Nile red stock solution (1 mg/ml) was prepared as previously described and used at a 1:100 dilution (Greenspan et al., 1985). Nile Red was visualized at an excitation wavelength of 480 nm and emission wavelength of >530 nm. Oil Red O sample images were analyzed in ImageJ by a blinded experimenter. The width of a lipid droplet was measured at its widest point and binned into large (>160 μm), medium (125-160 μm) or small (<125 μm) droplets.

### Assessment of starvation resistance

Adipose cells of the larval fat body degenerate and are replaced by adult adipose cells by 3 days post-eclosion (Butterworth et al., 1965). To test adult resistance to starvation, virgin female adults were collected and incubated at 25°C for 3 days. Animals were then anesthetized with CO_2_, washed in PBS, and separated into vials with a PBS-soaked cotton ball (20 flies per vial). Flies were maintained at room temperature and assessed for mortality every 3 h.

### Statistics

All statistical analyses were performed in R v3.1.0. Chi-square analyses were performed using Yates and corrected for continuity. Fisher's exact test, one-way ANOVA, and *t*-test (two tailed, equal variance) were used as indicated in figure legends. Post hoc analysis of ANOVA was performed with Tukey HSD. Post hoc analysis of Chi-square and Fisher's exact tests used the Bonferroni correction when applicable. The following critical *P* values were used for all analyses prior to correction: 0.05, 0.01, and 0.001.

## Supplementary Material

Supplementary information

## References

[BIO035394C1] Ali Y. O., Escala W., Ruan K. and Zhai R. G. (2011). Assaying locomotor, learning, and memory deficits in Drosophila models of neurodegeneration. *J. Vis. Exp.* 49, e2504 10.3791/2504 PMC3197301 21445036

[BIO035394C2] Anreiter I., Vasquez O. E., Allen A. M. and Sokolowski M. B. (2016). Foraging path-length protocol for Drosophila melanogaster larvae. *J. Vis. Exp.* 110, e53980 10.3791/53980 PMC4941989 27167330

[BIO035394C3] Apostolopoulou A. A., Hersperger F., Mazija L., Widmann A., Wust A. and Thum A. S. (2014). Composition of agarose substrate affects behavioral output of Drosophila larvae. *Front. Behav. Neurosci.* 8, 11 10.3389/fnbeh.2014.00011 24478658 PMC3904111

[BIO035394C4] Arrese E. L. and Soulages J. L. (2010). Insect fat body: energy, metabolism, and regulation. *Annu. Rev. Entomol.* 55, 207-225. 10.1146/annurev-ento-112408-085356 19725772 PMC3075550

[BIO035394C5] Avila F. W. and Wolfner M. F. (2009). Acp36DE is required for uterine conformational changes in mated Drosophila females. *Proc. Natl. Acad. Sci. USA* 106, 15796-15800. 10.1073/pnas.0904029106 19805225 PMC2747198

[BIO035394C6] Avila F. W., Ravi Ram K., Bloch Qazi M. C. and Wolfner M. F. (2010). Sex peptide is required for the efficient release of stored sperm in mated Drosophila females. *Genetics* 186, 595-600. 10.1534/genetics.110.119735 20679516 PMC2954482

[BIO035394C7] Avila F. W., Sirot L. K., LaFlamme B. A., Rubinstein C. D. and Wolfner M. F. (2011). Insect seminal fluid proteins: identification and function. *Annu. Rev. Entomol.* 56, 21-40. 10.1146/annurev-ento-120709-144823 20868282 PMC3925971

[BIO035394C8] Berdougo E., Nachury M. V., Jackson P. K. and Jallepalli P. V. (2008). The nucleolar phosphatase Cdc14B is dispensable for chromosome segregation and mitotic exit in human cells. *Cell Cycle* 7, 1184-1190. 10.4161/cc.7.9.5792 18418058

[BIO035394C9] Brodsky M. H., Nordstrom W., Tsang G., Kwan E., Rubin G. M. and Abrams J. M. (2000). Drosophila p53 binds a damage response element at the reaper locus. *Cell* 101, 103-113. 10.1016/S0092-8674(00)80627-3 10778860

[BIO035394C10] Brody T. (1999). The Interactive Fly: gene networks, development and the Internet. *Trends Genet.* 15, 333-334. 10.1016/S0168-9525(99)01775-8 10431196

[BIO035394C11] Buffone M. G., Schindler K. and Schultz R. M. (2014). Over-expression of CDC14B causes mitotic arrest and inhibits zygotic genome activation in mouse preimplantation embryos. *Cell Cycle* 8, 3904-3913. 10.4161/cc.8.23.10074 PMC3188409 19923902

[BIO035394C12] Butterworth F. M., Bodenstein D. and King R. C. (1965). Adipose tissue of Drosophila melanogaster. I. An experimental study of larval fat body. *J. Exp. Zool.* 158, 141-153. 10.1002/jez.1401580203 14327184

[BIO035394C14] Cho H. P., Liu Y., Gomez M., Dunlap J., Tyers M. and Wang Y. (2005). The dual-specificity phosphatase CDC14B bundles and stabilizes microtubules. *Mol. Cell. Biol.* 25, 4541-4551. 10.1128/MCB.25.11.4541-4551.2005 15899858 PMC1140622

[BIO035394C15] Clément A., Solnica-Krezel L. and Gould K. L. (2011). The Cdc14B phosphatase contributes to ciliogenesis in zebrafish. *Development* 138, 291-302. 10.1242/dev.055038 21177342 PMC3005604

[BIO035394C16] Clément A., Solnica-Krezel L. and Gould K. L. (2012). Functional redundancy between Cdc14 phosphatases in zebrafish ciliogenesis. *Dev. Dyn.* 241, 1911-1921. 10.1002/dvdy.23876 23027426 PMC3508521

[BIO035394C17] Clemente-Blanco A., Mayán-Santos M., Schneider D. A., Machín F., Jarmuz A., Tschochner H. and Aragón L. (2009). Cdc14 inhibits transcription by RNA polymerase I during anaphase. *Nature* 458, 219-222. 10.1038/nature07652 19158678 PMC4445138

[BIO035394C18] Clemente-Blanco A., Sen N., Mayan-Santos M., Sacristán M. P., Graham B., Jarmuz A., Giess A., Webb E., Game L., Eick D. et al. (2011). Cdc14 phosphatase promotes segregation of telomeres through repression of RNA polymerase II transcription. *Nat. Cell Biol.* 13, 1450-1456. 10.1038/ncb2365 22020438 PMC3232454

[BIO035394C19] Clifford D. M., Wolfe B. A., Roberts-Galbraith R. H., McDonald W. H., Yates J. R. III and Gould K. L. (2008). The Clp1/Cdc14 phosphatase contributes to the robustness of cytokinesis by association with anillin-related Mid1. *J. Cell Biol.* 181, 79-88. 10.1083/jcb.200709060 18378776 PMC2287289

[BIO035394C20] Crowther D. C., Kinghorn K. J., Miranda E., Page R., Curry J. A., Duthie F. A. I., Gubb D. C. and Lomas D. A. (2005). Intraneuronal Abeta, non-amyloid aggregates and neurodegeneration in a Drosophila model of Alzheimer's disease. *Neuroscience* 132, 123-135. 10.1016/j.neuroscience.2004.12.025 15780472

[BIO035394C21] Cueille N., Salimova E., Esteban V., Blanco M., Moreno S., Bueno A. and Simanis V. (2001). Flp1, a fission yeast orthologue of the s. cerevisiae CDC14 gene, is not required for cyclin degradation or rum1p stabilisation at the end of mitosis. *J. Cell Sci.* 114, 2649-2664. 11683392 10.1242/jcs.114.14.2649

[BIO035394C22] Field L. H. and Matheson T. (1998). Chordotonal organs of insects. *Adv. Insect Physiol.* 27, 1-228. 10.1016/S0065-2806(08)60013-2

[BIO035394C23] Figueroa-Clarevega A. and Bilder D. (2015). Malignant Drosophila tumors interrupt insulin signaling to induce cachexia-like wasting. *Dev. Cell* 33, 47-55. 10.1016/j.devcel.2015.03.001 25850672 PMC4390765

[BIO035394C24] Fisher K. H., Wright V. M., Taylor A., Zeidler M. P. and Brown S. (2012). Advances in genome-wide RNAi cellular screens: a case study using the Drosophila JAK/STAT pathway. *BMC Genomics* 13, 506 10.1186/1471-2164-13-506 23006893 PMC3526451

[BIO035394C25] Forbes A. and Lehmann R. (1998). Nanos and Pumilio have critical roles in the development and function of Drosophila germline stem cells. *Development* 125, 679-690. 9435288 10.1242/dev.125.4.679

[BIO035394C26] Gong W. J. and Golic K. G. (2003). Ends-out, or replacement, gene targeting in Drosophila. *Proc. Natl. Acad. Sci. USA* 100, 2556-2561. 10.1073/pnas.0535280100 12589026 PMC151379

[BIO035394C27] Gramates L. S., Marygold S. J., dos Santos G., Urbano J.-M., Antonazzo G., Matthews B. B., Rey A. J., Tabone C. J., Crosby M. A., Emmert D. B. et al. (2017). FlyBase at 25: looking to the future. *Nucleic Acids Res.* 45, D663-D671. 10.1093/nar/gkw1016 27799470 PMC5210523

[BIO035394C28] Greenspan R. J. (2004). *Fly Pushing: The Theory and Practice of Drosophila Genetics*. Cold Spring Harbor, NY: Cold Spring Harbor Laboratory Press.

[BIO035394C29] Greenspan P., Mayer A. P. and Fowler S. D. (1985). Nile red: a selective fluorescent stain for intracellular lipid droplets. *J. Cell Biol.* 100, 965-973. 10.1083/jcb.100.3.965 3972906 PMC2113505

[BIO035394C30] Guillamot M., Manchado E., Chiesa M., Gómez-López G., Pisano D. G., Sacristán M. P. and Malumbres M. (2011). Cdc14b regulates mammalian RNA polymerase II and represses cell cycle transcription. *Sci. Rep.* 1, 189 10.1038/srep00189 22355704 PMC3240995

[BIO035394C31] Gutierrez E., Wiggins D., Fielding B. and Gould A. P. (2007). Specialized hepatocyte-like cells regulate Drosophila lipid metabolism. *Nature* 445, 275-280. 10.1038/nature05382 17136098

[BIO035394C32] Hainline S. G., Rickmyre J. L., Neitzel L. R., Lee L. A. and Lee E. (2014). The Drosophila MCPH1-B isoform is a substrate of the APCCdh1 E3 ubiquitin ligase complex. *Biol. Open* 3, 669-676. 10.1242/bio.20148318 24972868 PMC4154303

[BIO035394C33] Huey R. B., Suess J., Hamilton H. and Gilchrist G. W. (2004). Starvation resistance in Drosophila melanogaster: testing for a possible ‘cannibalism’ bias. *Funct. Ecol.* 18, 952-954. 10.1111/j.0269-8463.2004.00915.x

[BIO035394C34] Jang K. P., Jang T. and Davidowitz G. (2014). Exploring the nutritional basis of starvation resistance in Drosophila melanogaster. *Funct. Ecol.* 28, 1144-1155. 10.1111/1365-2435.12247

[BIO035394C35] Kaiser B. K., Nachury M. V., Gardner B. E. and Jackson P. K. (2004). Xenopus Cdc14 alpha/beta are localized to the nucleolus and centrosome and are required for embryonic cell division. *BMC Cell Biol.* 5, 27 10.1186/1471-2121-5-27 15251038 PMC481057

[BIO035394C36] Keil T. A. (1997). Functional morphology of insect mechanoreceptors. *Microsc. Res. Tech.* 39, 506-531. 10.1002/(SICI)1097-0029(19971215)39:6<506::AID-JEMT5>3.0.CO;2-B 9438251

[BIO035394C37] Kernan M., Cowan D. and Zuker C. (1994). Genetic dissection of mechanosensory transduction: mechanoreception-defective mutations of Drosophila. *Neuron* 12, 1195-1206. 10.1016/0896-6273(94)90437-5 8011334

[BIO035394C38] Kobayashi S., Yamada M., Asaoka M. and Kitamura T. (1996). Essential role of the posterior morphogen nanos for germline development in Drosophila. *Nature* 380, 708-711. 10.1038/380708a0 8614464

[BIO035394C39] Krasinska L., de Bettignies G., Fisher D., Abrieu A., Fesquet D. and Morin N. (2007). Regulation of multiple cell cycle events by Cdc14 homologues in vertebrates. *Exp. Cell Res.* 313, 1225-1239. 10.1016/j.yexcr.2006.12.022 17292885

[BIO035394C40] Laurençon A., Dubruille R., Efimenko E., Grenier G., Bissett R., Cortier E., Rolland V., Swoboda P. and Durand B. (2007). Identification of novel regulatory factor X (RFX) target genes by comparative genomics in Drosophila species. *Genome Biol.* 8, R195 10.1186/gb-2007-8-9-r195 17875208 PMC2375033

[BIO035394C41] Lee E., Sivan-Loukianova E., Eberl D. F. and Kernan M. J. (2008). An IFT-A protein is required to delimit functionally distinct zones in mechanosensory cilia. *Curr. Biol.* 18, 1899-1906. 10.1016/j.cub.2008.11.020 19097904 PMC2615538

[BIO035394C42] Li L., Ljungman M. and Dixon J. E. (2000). The human Cdc14 phosphatases interact with and dephosphorylate the tumor suppressor protein p53. *J. Biol. Chem.* 275, 2410-2414. 10.1074/jbc.275.4.2410 10644693

[BIO035394C13] Lilly M. and Carlson J. (1990). Smellblind - a Gene Required for Drosophila Olfaction. *Genetics* 124, 293-302. 2106470 10.1093/genetics/124.2.293 PMC1203922

[BIO035394C43] Liu X., Jiang F., Kalidas S., Smith D. and Liu Q. (2006). Dicer-2 and R2D2 coordinately bind siRNA to promote assembly of the siRISC complexes. *RNA* 12, 1514-1520. 10.1261/rna.101606 16775303 PMC1524895

[BIO035394C44] Ma L. and Jarman A. P. (2011). Dilatory is a Drosophila protein related to AZI1 (CEP131) that is located at the ciliary base and required for cilium formation. *J. Cell Sci.* 124, 2622-2630. 10.1242/jcs.084798 21750193 PMC3138703

[BIO035394C45] Machin F., Quevedo O., Ramos-Perez C. and Garcia-Luis J. (2016). Cdc14 phosphatase: warning, no delay allowed for chromosome segregation! *Curr. Genet.* 62, 7-13. 10.1007/s00294-015-0502-1 26116076 PMC4723626

[BIO035394C76] Maggert K. A., Gong W. J. and Golic K. G. (2008). Methods for homologous recombination in Drosophila. *Methods Mol. Biol.* 420, 155-174. 10.1007/978-1-59745-583-1_9 18641946

[BIO035394C46] Mocciaro A. and Schiebel E. (2010). Cdc14: a highly conserved family of phosphatases with non-conserved functions? *J Cell Sci.* 123, 2867-2876. 10.1242/jcs.074815 20720150

[BIO035394C47] Mocciaro A., Berdougo E., Zeng K., Black E., Vagnarelli P., Earnshaw W., Gillespie D., Jallepalli P. and Schiebel E. (2010). Vertebrate cells genetically deficient for Cdc14A or Cdc14B retain DNA damage checkpoint proficiency but are impaired in DNA repair. *J. Cell Biol.* 189, 631-639. 10.1083/jcb.200910057 20479464 PMC2872905

[BIO035394C48] Nichols C. D. (2015). *Life Extension Lessons from Drosophila*. Switzerland: Springer.

[BIO035394C49] Nichols C. D., Becnel J. and Pandey U. B. (2012). Methods to assay Drosophila behavior. *J. Vis. Exp.* 61, e3795 10.3791/3795 PMC3671839 22433384

[BIO035394C50] Papadopoulou K., Chen J.-S., Mead E., Feoktistova A., Petit C., Agarwal M., Jamal M., Malik A., Spanos A., Sedgwick S. G. et al. (2010). Regulation of cell cycle-specific gene expression in fission yeast by the Cdc14p-like phosphatase Clp1p. *J. Cell Sci.* 123, 4374-4381. 10.1242/jcs.073056 21098641

[BIO035394C51] Peng J., Chen S., Büsser S., Liu H., Honegger T. and Kubli E. (2005). Gradual release of sperm bound sex-peptide controls female postmating behavior in Drosophila. *Curr. Biol.* 15, 207-213. 10.1016/j.cub.2005.01.034 15694303

[BIO035394C52] Pospisilik J. A., Schramek D., Schnidar H., Cronin S. J. F., Nehme N. T., Zhang X., Knauf C., Cani P. D., Aumayr K., Todoric J. et al. (2010). Drosophila genome-wide obesity screen reveals hedgehog as a determinant of brown versus white adipose cell fate. *Cell* 140, 148-160. 10.1016/j.cell.2009.12.027 20074523

[BIO035394C53] Price C. S. C., Dyer K. A. and Coyne J. A. (1999). Sperm competition between Drosophila males involves both displacement and incapacitation. *Nature* 400, 449-452. 10.1038/22755 10440373

[BIO035394C54] Purves D. C. and Brachmann C. (2007). Dissection of imaginal discs from 3rd instar Drosophila larvae. *J Vis Exp*, 140 10.3791/140 18830432 PMC2532944

[BIO035394C55] Queralt E. and Uhlmann F. (2008). Separase cooperates with Zds1 and Zds2 to activate Cdc14 phosphatase in early anaphase. *J. Cell Biol.* 182, 873-883. 10.1083/jcb.200801054 18762578 PMC2528575

[BIO035394C56] Riparbelli M. G., Callaini G. and Megraw T. L. (2012). Assembly and persistence of primary cilia in dividing Drosophila spermatocytes. *Dev. Cell* 23, 425-432. 10.1016/j.devcel.2012.05.024 22898783 PMC3422508

[BIO035394C57] Rodier G., Coulombe P., Tanguay P.-L., Boutonnet C. and Meloche S. (2008). Phosphorylation of Skp2 regulated by CDK2 and Cdc14B protects it from degradation by APC(Cdh1) in G1 phase. *EMBO J.* 27, 679-691. 10.1038/emboj.2008.6 18239684 PMC2262036

[BIO035394C58] Rogers D. W., Baldini F., Battaglia F., Panico M., Dell A., Morris H. R. and Catteruccia F. (2009). Transglutaminase-mediated semen coagulation controls sperm storage in the malaria mosquito. *PLoS Biol.* 7, e1000272 10.1371/journal.pbio.1000272 20027206 PMC2785878

[BIO035394C59] Rørth P. (1998). Gal4 in the Drosophila female germline. *Mech. Dev.* 78, 113-118. 10.1016/S0925-4773(98)00157-9 9858703

[BIO035394C60] Rosso L., Marques A. C., Weier M., Lambert N., Lambot M.-A., Vanderhaeghen P. and Kaessmann H. (2008). Birth and rapid subcellular adaptation of a hominoid-specific CDC14 protein. *PLoS Biol.* 6, e140 10.1371/journal.pbio.0060140 18547142 PMC2422853

[BIO035394C61] Rubin G. M. and Spradling A. C. (1982). Genetic transformation of Drosophila with transposable element vectors. *Science* 218, 348-353. 10.1126/science.6289436 6289436

[BIO035394C62] Rüthnick D. and Schiebel E. (2016). Duplication of the Yeast Spindle Pole Body Once per Cell Cycle. *Mol. Cell. Biol.* 36, 1324-1331. 10.1128/MCB.00048-16 26951196 PMC4836218

[BIO035394C63] Saito R. M., Perreault A., Peach B., Satterlee J. S. and van den Heuvel S. (2004). The CDC-14 phosphatase controls developmental cell-cycle arrest in C. elegans. *Nat. Cell Biol.* 6, 777-783. 10.1038/ncb1154 15247923

[BIO035394C64] Sarkissian T., Timmons A., Arya R., Abdelwahid E. and White K. (2014). Detecting apoptosis in Drosophila tissues and cells. *Methods* 68, 89-96. 10.1016/j.ymeth.2014.02.033 24613678 PMC4048790

[BIO035394C65] Shim J., Lee Y., Jeong Y. T., Kim Y., Lee M. G., Montell C. and Moon S. J. (2015). The full repertoire of Drosophila gustatory receptors for detecting an aversive compound. *Nat. Commun.* 6, 8867 10.1038/ncomms9867 26568264 PMC4660205

[BIO035394C66] Sitaram P., Hainline S. G. and Lee L. A. (2014). Cytological analysis of spermatogenesis: live and fixed preparations of Drosophila testes. *J. Vis. Exp.* 83, e51058 10.3791/51058 PMC4089415 24473184

[BIO035394C67] Stapleton M., Liao G., Brokstein P., Hong L., Carninci P., Shiraki T., Hayashizaki Y., Champe M., Pacleb J., Wan K. et al. (2002). The Drosophila gene collection: identification of putative full-length cDNAs for 70% of D. melanogaster genes. *Genome Res.* 12, 1294-1300. 10.1101/gr.269102 12176937 PMC186637

[BIO035394C68] Stegmeier F. and Amon A. (2004). Closing mitosis: the functions of the Cdc14 phosphatase and its regulation. *Annu. Rev. Genet.* 38, 203-232. 10.1146/annurev.genet.38.072902.093051 15568976

[BIO035394C69] Stewart S., Koh T.-W., Ghosh A. C. and Carlson J. R. (2015). Candidate ionotropic taste receptors in the Drosophila larva. *Proc. Natl. Acad. Sci. USA* 112, 4195-4201. 10.1073/pnas.1503292112 25825777 PMC4394268

[BIO035394C70] Wang C. and Lehmann R. (1991). Nanos is the localized posterior determinant in drosophila. *Cell* 66, 637-647. 10.1016/0092-8674(91)90110-K 1908748

[BIO035394C71] Wolfe B. A. and Gould K. L. (2004). Fission yeast Clp1p phosphatase affects G2/M transition and mitotic exit through Cdc25p inactivation. *EMBO J.* 23, 919-929. 10.1038/sj.emboj.7600103 14765109 PMC381010

[BIO035394C72] Wu Q., Zhao Z. W. and Shen P. (2005). Regulation of aversion to noxious food by Drosophila neuropeptide Y- and insulin-like systems. *Nat. Neurosci.* 8, 1350-1355. 10.1038/nn1540 16172603

[BIO035394C73] Wu J., Cho H. P., Rhee D. B., Johnson D. K., Dunlap J., Liu Y. and Wang Y. (2008). Cdc14B depletion leads to centriole amplification, and its overexpression prevents unscheduled centriole duplication. *J. Cell Biol.* 181, 475-483. 10.1083/jcb.200710127 18458157 PMC2364701

[BIO035394C74] Yeh S.-D., Chan C. and Ranz J. M. (2013). Assessing differences in sperm competitive ability in Drosophila. *J. Vis. Exp.* 78, e50547 10.3791/50547 PMC3856288 23995693

[BIO035394C75] Zamore P. D. and Ma S. (2011). Isolation of Drosophila melanogaster testes. *J. Vis. Exp.* 51, e2641 10.3791/2641 PMC3197105 21610676

