# Correction: Characterization of a *cdc14* null allele in *Drosophila melanogaster* (doi:10.1242/bio.035394)

**DOI:** 10.1242/bio.037705

**Published:** 2018-08-15

**Authors:** Leif Neitzel, Matthew Broadus, Nailing Zhang, Leah Sawyer, Heather Wallace, Julie Merkle, Jeanne Jodoin, Poojitha Sitaram, Emily Crispi, William Rork, Laura Lee, Duojia Pan, Kathleen Gould, Andrea Page-McCaw, Ethan Lee

There were errors published in Biology Open 2018 7: bio035394 doi:10.1242/bio.035394

Incorrect versions of Figure 2B and Figure S5B were used for the published version of this article.

The corrected figures are shown below.

**Fig. 2.**
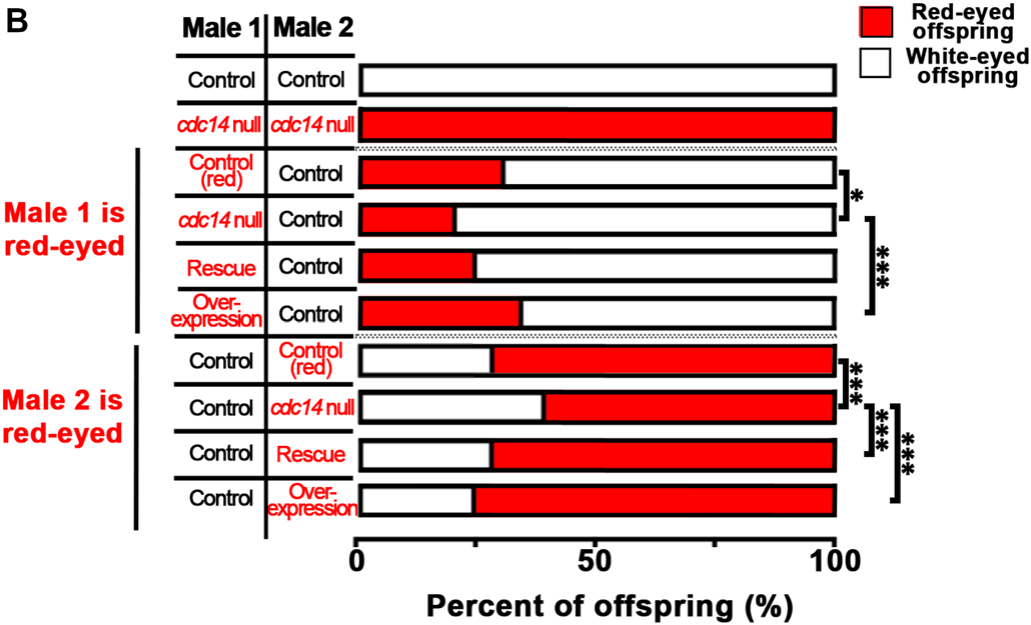
***cdc14*** **null males exhibit decreased sperm competition.** (B) A control experiment was performed using white-eyed *y w* males for both the first and second males. A second control experiment was performed using red-eyed *cdc14* null males for both the first and second males. The *cdc14* null males are less competitive compared to control males regardless of whether they are the first or second male to mate. Results for a single representative replicates (*n*≥15 vials per cross) are shown. Additional data can be found in Fig. S5B. Data were analyzed using a Chi-squared test with Bonferroni correction. Six pairwise comparisons were made. Red-eyed control males were compared to the *cdc14* null, rescue, or overexpression males; *cdc14* null males were compared to rescue or overexpression male; and rescue males were compared to overexpression males. *P<0.009. ***P<0.0002.

**Figure S5.**
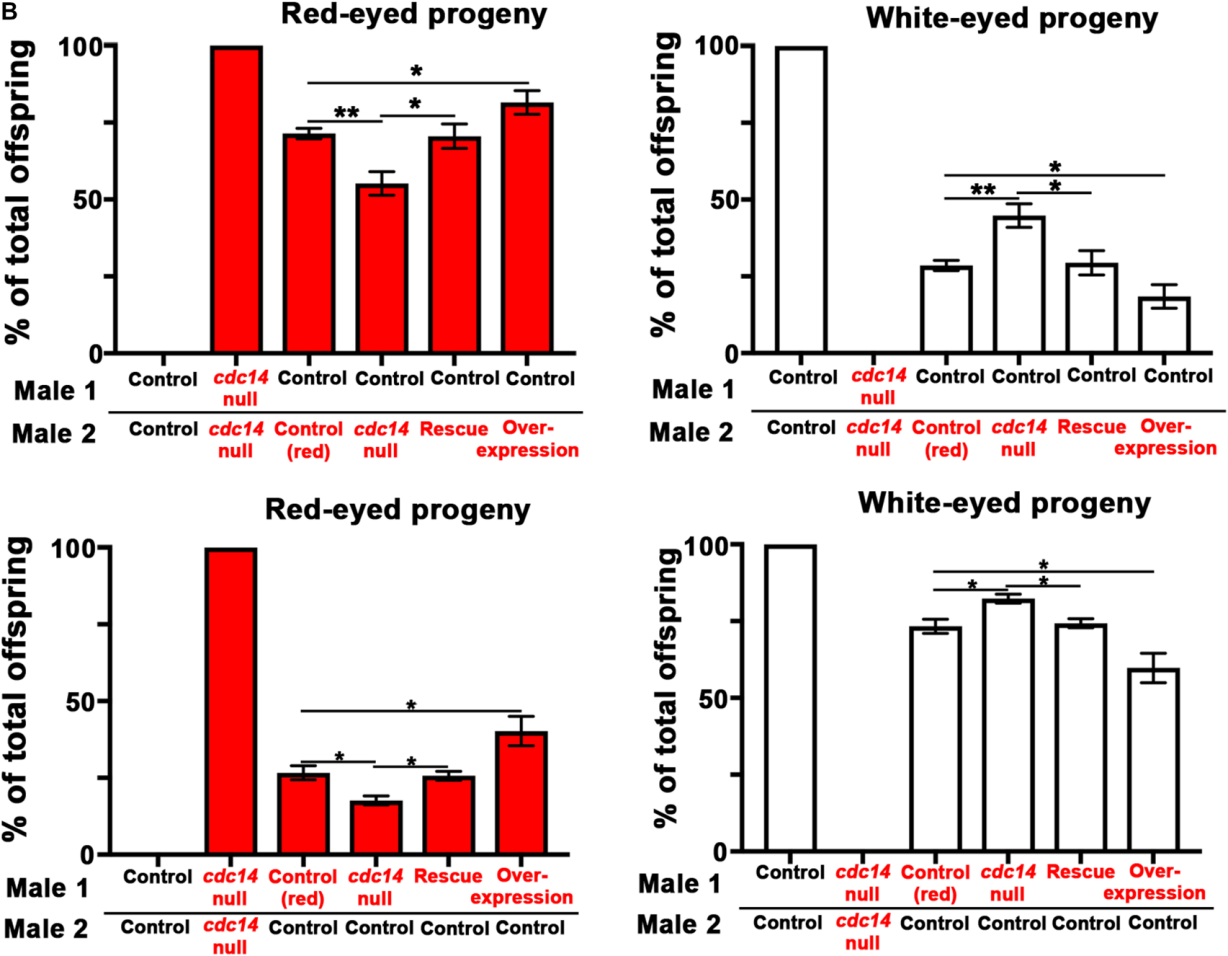
**cdc14 null males mate overnight at the same rate as controls.** (B) Eye color of offspring from all replicates of the sperm competition assay. The proportion of offspring from *cdc14* null males was significantly lower than the control (red). This decrease was rescued by expression of *nos*>*myc-cdc14*. Data were analyzed by Chi-squared test with Bonferroni correction. Control (red) was compared to the *cdc14* null, rescue, and overexpression. The cdc14 null was compared to the rescue. *ρ<0.02, **ρ<0.003, ***ρ<0.0003. In (A) and (B), N≥48 vials aggregated in N≥3 experiments.

None of these errors affected the conclusions of the paper.

The authors apologize for any inconvenience caused by these changes.

