# Supplementary material for: Characterization of a cdc14 null allele in Drosophila melanogaster
Source: Biol Open. 2018 Jun 26;7(7):bio035394. doi: 10.1242/bio.035394 (PMC6078348; doi:10.1242/bio.035394)
Supplement: Supplementary information [file biolopen-7-035394-s1.pdf]

Table S1. Representative metazoan Cdc14 orthologs identified across phyla.

Species	Cdc14 Orthologs
S. cerevisiae	*CDC14*
S. pombe	*clp1/flp1*
C. elegans	*cdc-14*
D. melanogaster	*cdc14*
D. rerio	*cdc14A,cdc14B*
X. laevis	*cdc14A,cdc14B*
G. gallus	*CDC14A,CDC14B*
M. musculus	*Cdc14A,Cdc14B*
H. sapiens	*CDC14A,CDC14B,CDC14C*

Biology Open • Supplementary information

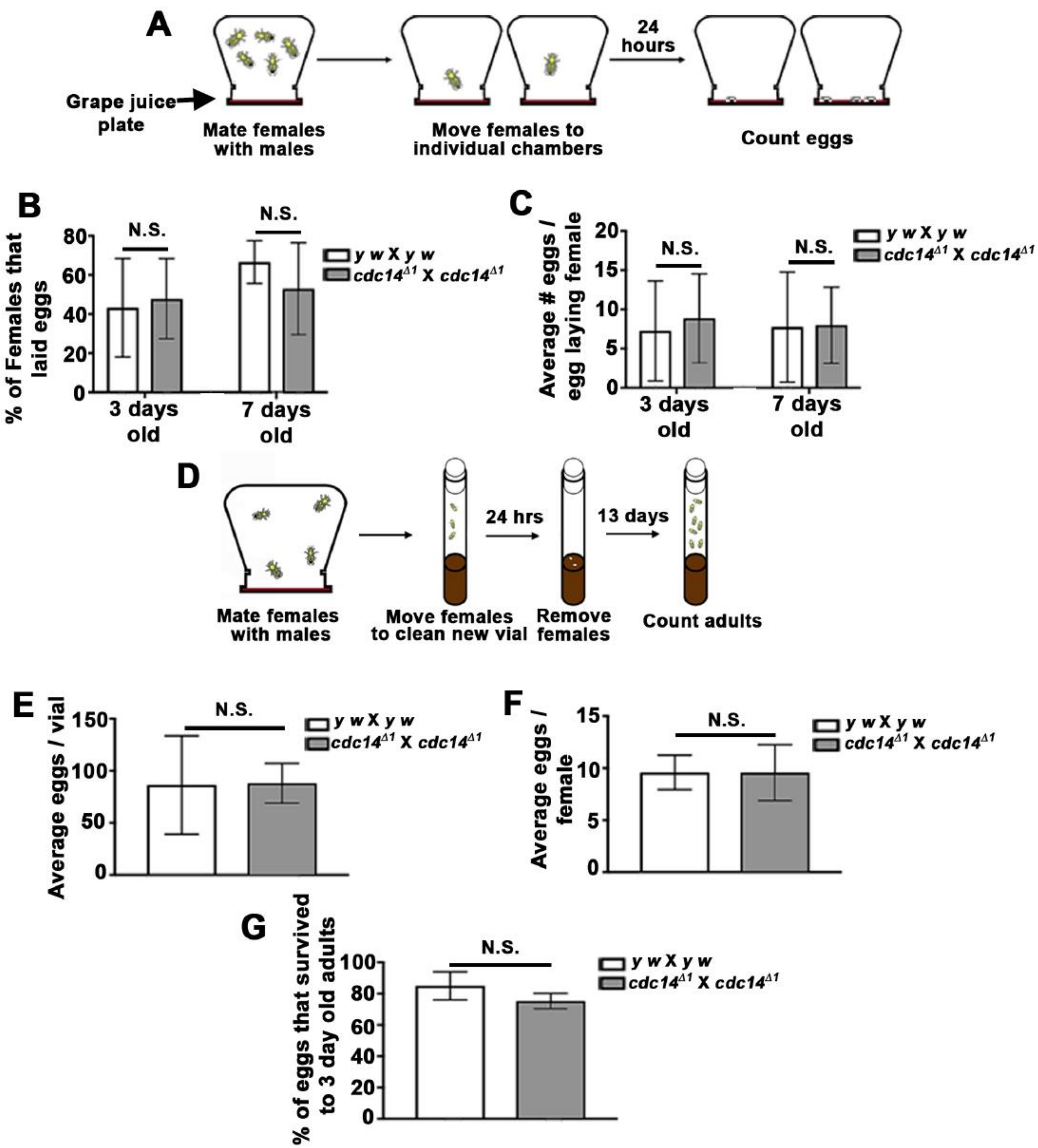

Figure S1. *cdc14* is not required for female fertility or animal development. (A) Three or seven days old females were placed in individual egg laying chambers and

Biology Open • Supplementary information

allowed to lay eggs for 24 h. (B,C) No significant differences in the percentage of females laying eggs (B) or the number of embryos laid per female (C) were observed. Only females that laid eggs were included in the analysis in (C). Results were aggregated from females (N≥53) in N≥3 repetitions in (A), (B), and (C). (D) Ten females per vial were allowed to lay for 24 h. Females were then removed, and the number of eggs in each vial were counted and compared between vials (E) and by the total number of females (F). No differences in the number of eggs per vial (E) or eggs per female (F) were observed. (G) Eggs were allowed to hatch and larvae grown to adulthood. No differences in the number of eggs that survived to three days old adult animals were observed. Eggs (N≥200) were assessed from N=5 vials from N=3 repetitions in (E), (F), and (G). In (B), (C), (E), (F), and (G) both parental lines were *cdc14* nulls (*cdc14*$^{\Delta1}$/*cdc14*$^{\Delta1}$), and the *cdc14* nulls were compared to their genetic background line (*y w*). Results shown in (B), (C), (E), (F), and (G) were analyzed by Chi-squared test.

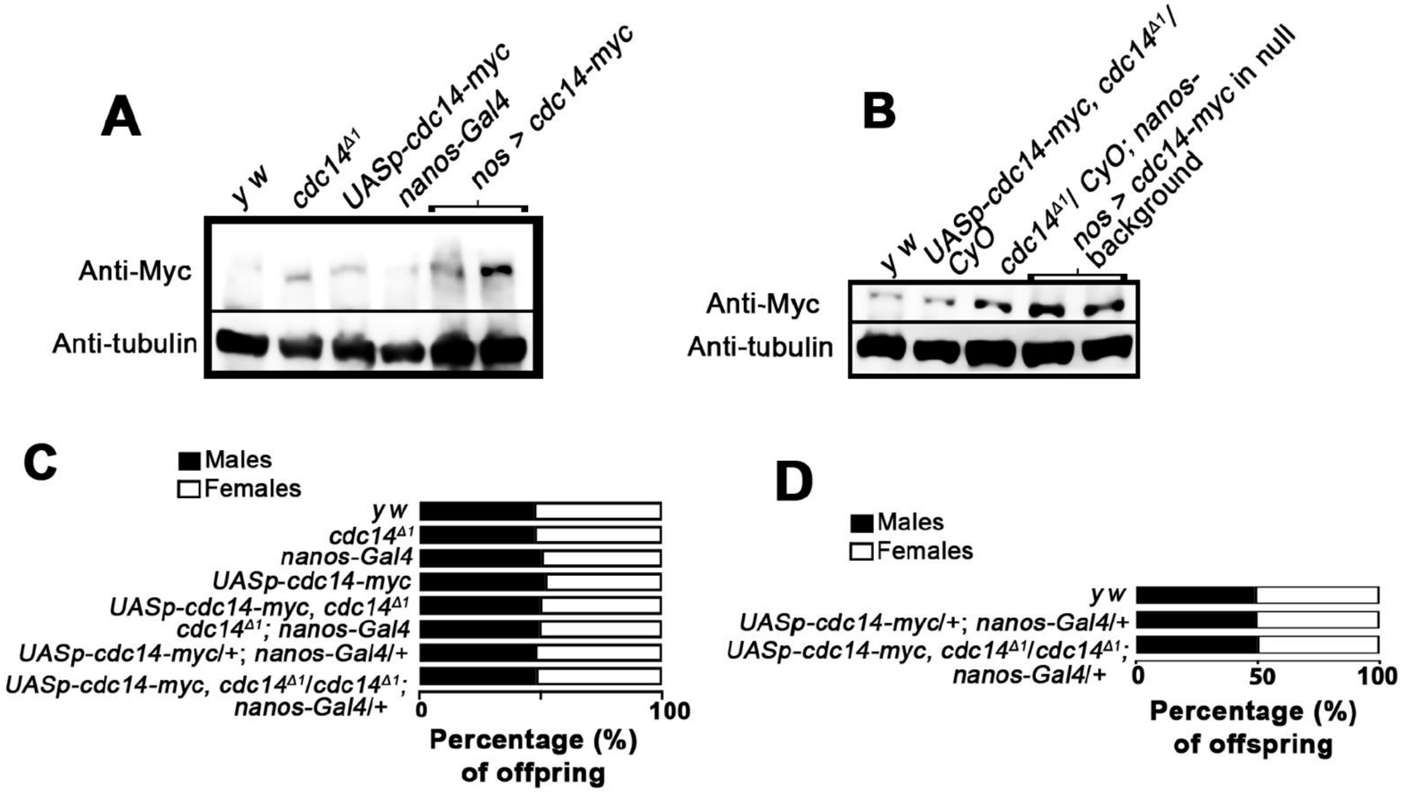

Figure S2. *cdc14* does not affect the ratio of male-to-female offspring, and *nos-gal4 > UASp-cdc14-myc* results in Cdc14-Myc protein expression. (A) Immunoblot analysis of Cdc14-Myc levels in third instar larvae expressing *UASp-cdc14-myc* under the control of *nanos-Gal4* (*nos-Gal4*) in a *y w* background. (B) Immunoblot analysis of Cdc14-Myc levels in third instar larvae expressing *UASp-cdc14-myc* under the control of *nos-Gal4* in a *cdc14* null background. (A) and (B) are representative gels of N=3 replicates. Tubulin was used as loading control. (C) The ratio of male to female offspring for lines used in this paper. Comparisons of different parental genotypes show no differences in the ratios of male to female offspring. Results were aggregated from offspring (N≥261) in N≥3 replicates. (D) Male offspring from the cross in (C) mated to a *y w* control female. Comparisons of male offspring from the different parental genotypes show no differences in the ratios of male to female offspring. Results were aggregated from offspring (N≥121) in N≥3 replicates.

Biology Open • Supplementary information

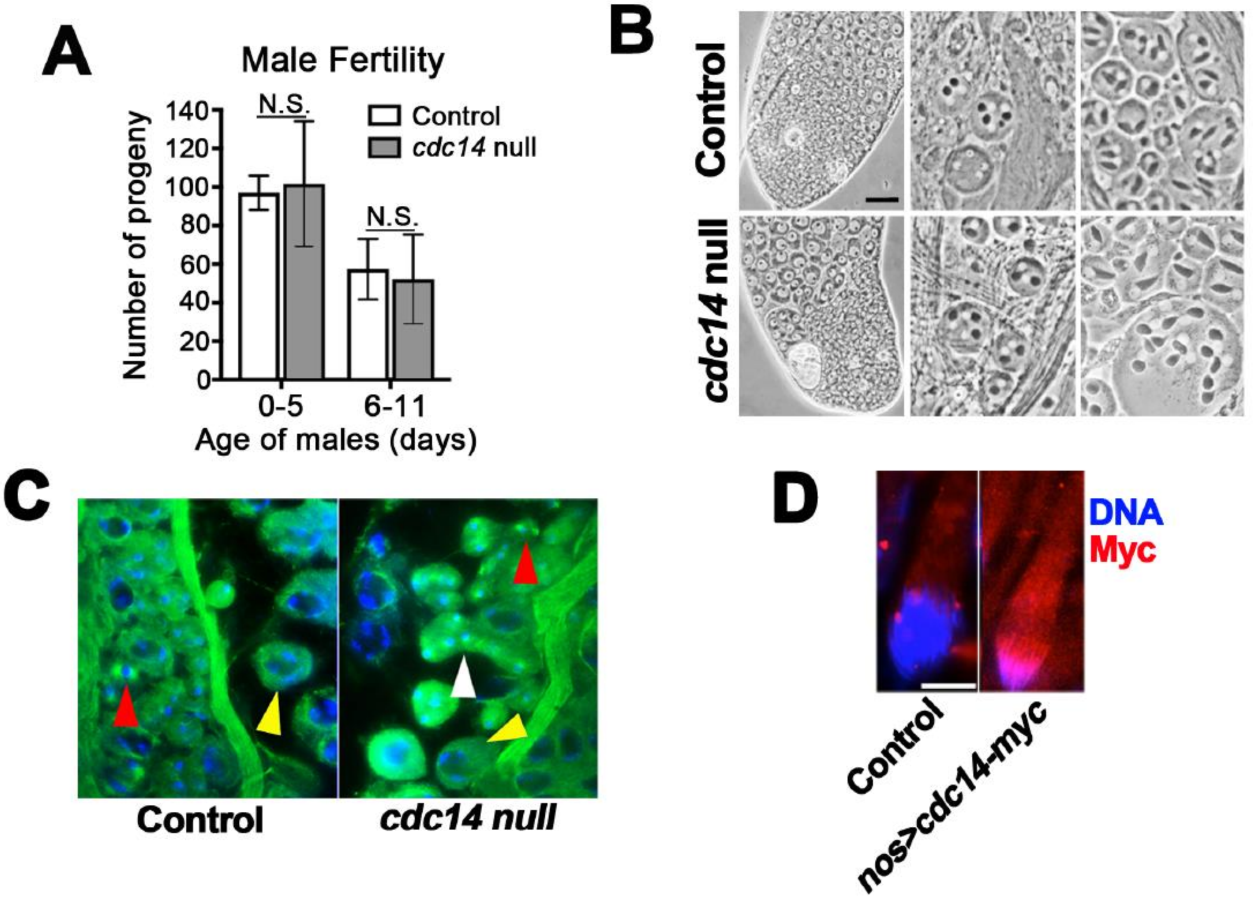

Figure S3. Cdc14-Myc localizes to the sperm bundle heads, but loss of *cdc14* does not affect male fertility or spermatid/sperm number or result in overt morphological changes of the testis. (A) Comparison of the number of progeny from a single control female crossed to 0-5 or 6-11 days old *cdc14* null versus control males. Results were analyzed by T-test (two tailed, equal variance; $\rho>0.05$). N.S. = not

Biology Open • Supplementary information

significant. Animals (N≥782) from N=15 crosses were assessed in (A). (B) and (C) Testes mutant for *cdc14* appear to exhibit normal spermatogenesis and cell divisions. (B) Bright-field images of control and *cdc14* null testes showed no consistent or significant gross morphological differences between groups. Three separate cross sections from different animals are shown. Left panels display early 2-16 cell stage spermatogonial cysts. The smaller cells are part of 2-4 cell cysts, whereas the larger cells are part of 8-16 cell cysts. Middle panels display round onion-stage spermatids. The right panels show early onion stage elongating spermatids that will form mature sperm. (C) Testes stained for tubulin (green) and DNA (blue, DAPI) showed no overt differences between *cdc14* null and control flies. Panels show a mixture of primary (yellow arrowheads) and secondary (red arrowheads) spermatocytes. The latter are shown undergoing and completing the second meiotic division. White arrowhead marks clearly observed early onion stage spermatids in the *cdc14* null testes. (D) Sperm bundles from control and *nos-Gal4 > cdc14-myc* male flies show localization of Cdc14-Myc to the head of the sperm bundle. Red is Myc and blue (DAPI) is DNA. Magnification, 100x. (B-D) are representative figures from N≥20 testes from N≥6 replicates. Scale bars, 10 µm. Testes from *y w* and *cdc14* null males were assessed for number of spermatids in the spermatogenic cyst (E) and number of mature sperm per bundle (F). No significant differences were observed in the number of spermatids (N=3 cysts from N=3 males in (E)) or sperm (N=3 mature sperm bundles from N=3 males in (F)). Results shown in (E) and (F) were analyzed by Chi-squared test.

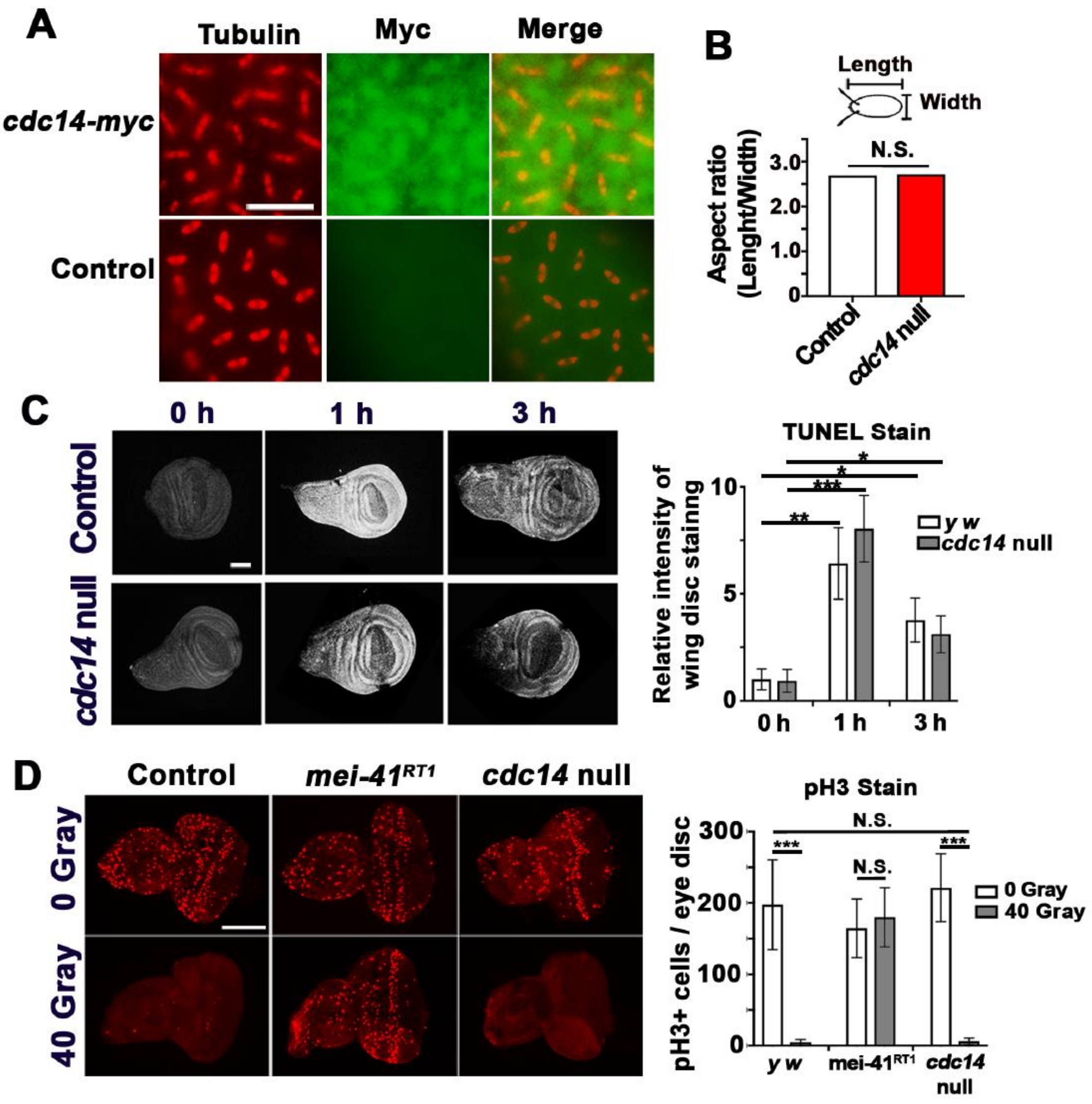

Figure S4. Cdc14-Myc is diffusely localized in the embryo and is not required for embryogenesis, apoptosis, or DNA damage repair. (A) Precellularized 0-2 h embryos showed diffuse localization of Cdc14-Myc that had been expressed under the control of *nanos-Gal4*. Tubulin is red and Myc is green. Magnification, 40x. Scale bar,

Biology Open • Supplementary information

20 μm. Embryos (N≥75) from N=2 replicates. (B) The ratio of the length to width of 1 hr post fertilization embryos showed no difference between control and *cdc14* null embryos. Results were analyzed by t-test (two tailed, equal variance). Eggs (N≥61) were aggregated from N≥3 replicates were assessed. (C) TUNEL-stained wing discs showed no difference between control and *cdc14* null third instar larvae upon exposure to ionizing radiation (40 Gray). Scale bars, 50 µm. Images were assessed in Fiji by measuring fluorescence of the entire sample. Treatments were normalized to the *y w* 0 h control. 0, 1, and 3 h time points were compared to each other as well as control to *cdc14* null at each time point. Results were analyzed by t-test (two-tailed, equal variance with Bonferroni correction) (Wing discs (N≥7) from N≥3 replicates). (D) Images of eye discs from third instar larvae stained with anti-phospho-Histone H3 to mark cells in mitosis. No changes in the mitotic index were observed between the *cdc14* null and control line upon irradiation. As a control, the $mei\text{-}41^{RT1}$ eye discs (defective in DNA damage repair) continued to undergo mitosis. Scale bars, 50 µm. Comparisons were made between 0 and 40 Gray treatments between genotypes. Results were analyzed by t-test (two-tailed, equal variance with Bonferroni correction). Experiments were performed using N≥25 eye discs in N≥3 replicates. *$\rho<0.008$, **$\rho<0.002$, ***$\rho<0.0002$.

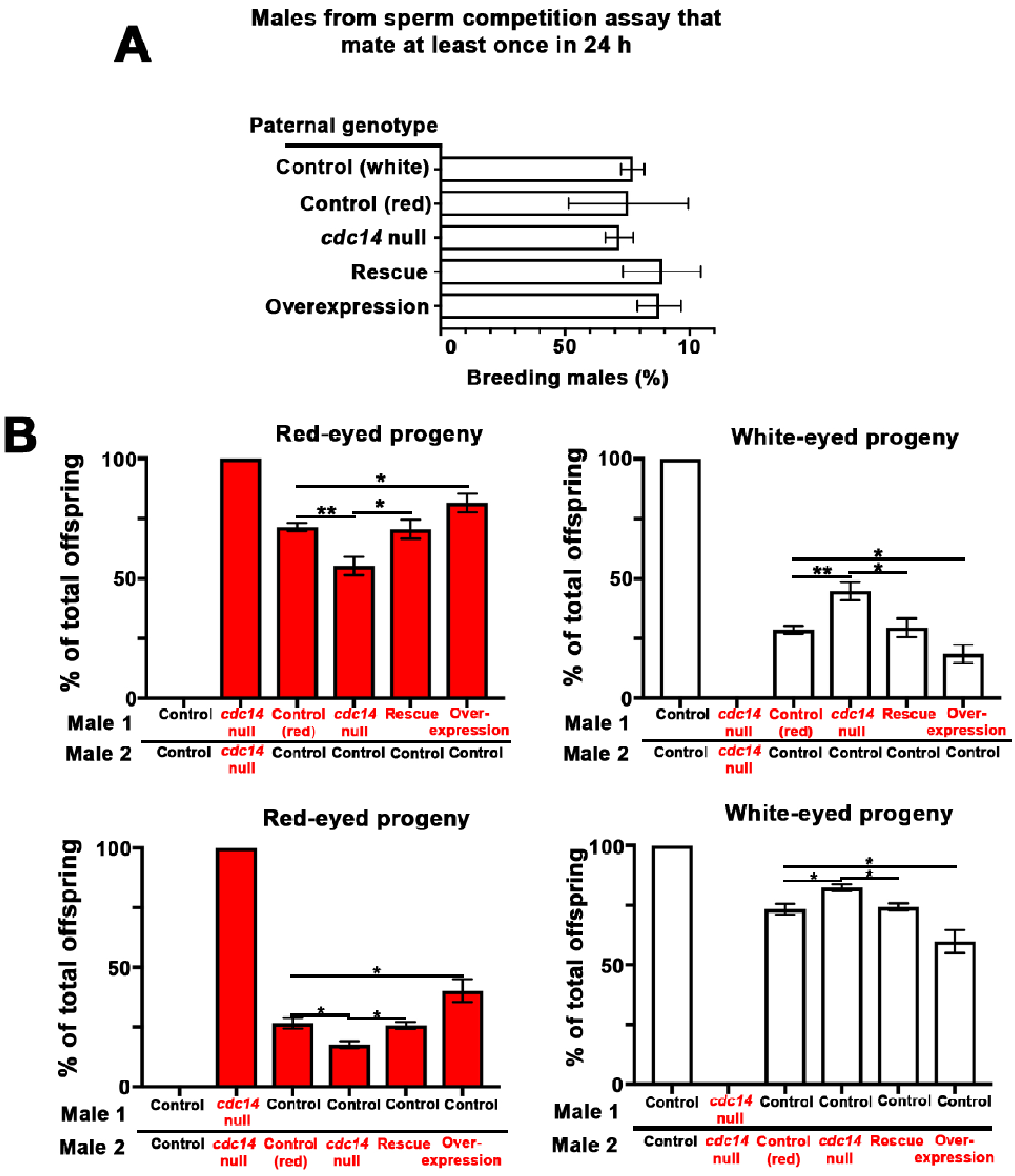

Biology Open • Supplementary information

Figure S5. *cdc14* null males mate overnight at the same rate as controls. (A) Vials from all sperm competition assays (see Figure 2) were assessed for progeny from each male. No significant differences were observed in the number of mating males of each genotype. Vials in which no progeny were observed for either the first or second male were excluded from all analyses of sperm competition. Data were analyzed by Chi-squared test with Bonferroni correction. (B) Eye color of offspring from all replicates of the sperm competition assay. The proportion of offspring from *cdc14* null males was significantly lower than the control (red). This decrease was rescued by expression of *nos* > *myc-cdc14*. Data were analyzed by Chi-squared test with Bonferroni correction. Control (red) was compared to the *cdc14* null, rescue, and overexpression. The *cdc14* null was compared to the rescue. * $\rho<0.02$, ** $\rho<0.003$, *** $\rho<0.0003$. In (A) and (B), N≥48 vials aggregated in N≥3 experiments.

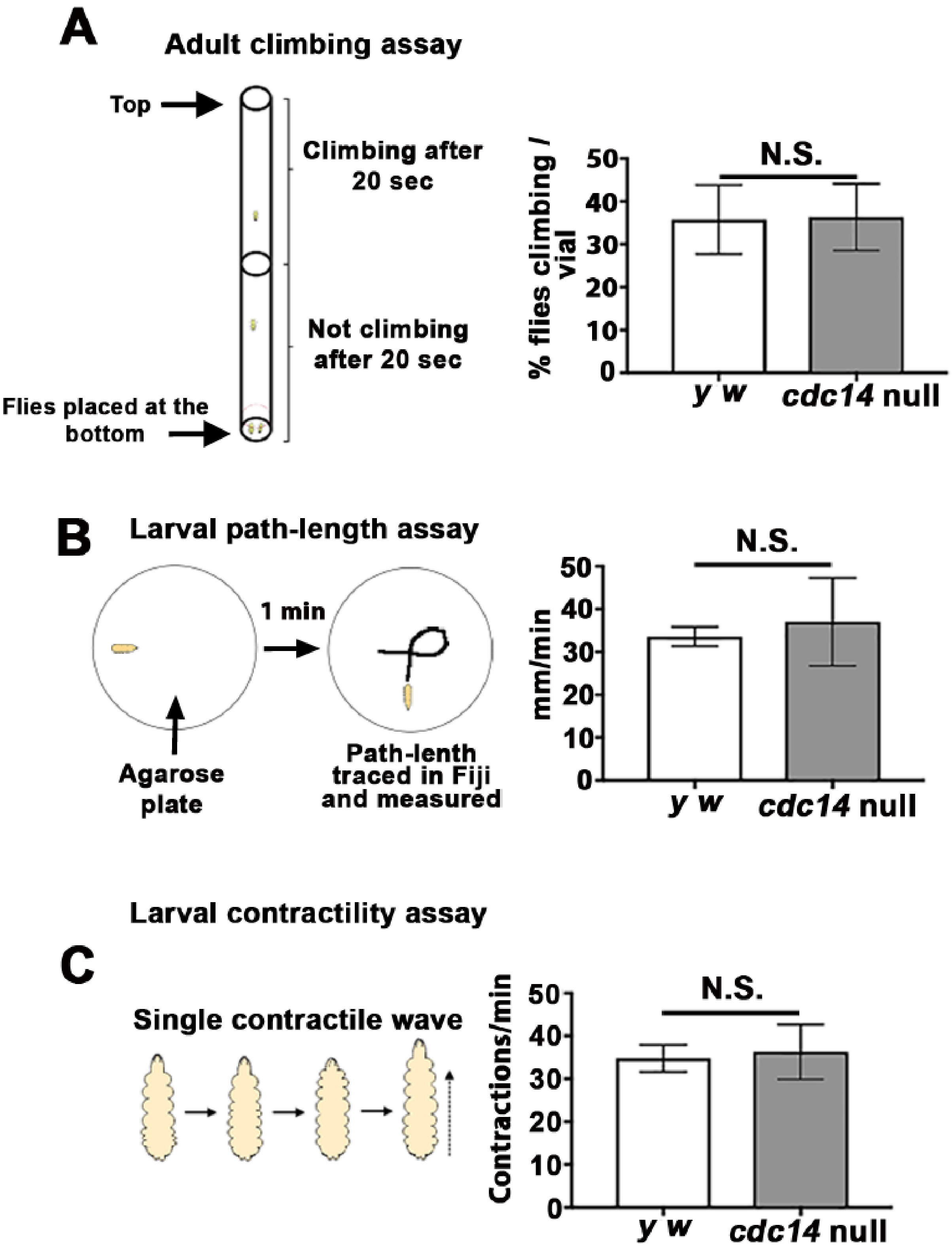

Biology Open • Supplementary information

Fig S6. *cdc14* nulls do not show impaired path-length or locomotion. (A) In the adult climbing assay (left), flies were allowed to climb for 20 seconds, followed by assessment of the number of flies in the top vial ("climbing" flies). The number of climbing adults was not significantly different between the control and *cdc14* null line. Results were aggregated from N>150 flies in N=3 replicates. (B) Third instar larvae were allowed to move freely for 1 min on an agarose plate (left). The larval path was traced and measured in Fiji. The average path-length traveled per larva per minute was not significantly different between the control and *cdc14* null line. (C) Larvae were observed under a brightfield microscope, and the number of full contractile waves (left) were counted for 1 min. No difference was observed between the control and *cdc14* null line. N=30 larvae were tested in (B) and (C). (A), (B), and (C) were analyzed by t-test (two tailed, equal variance; ρ>0.05).

Biology Open • Supplementary information

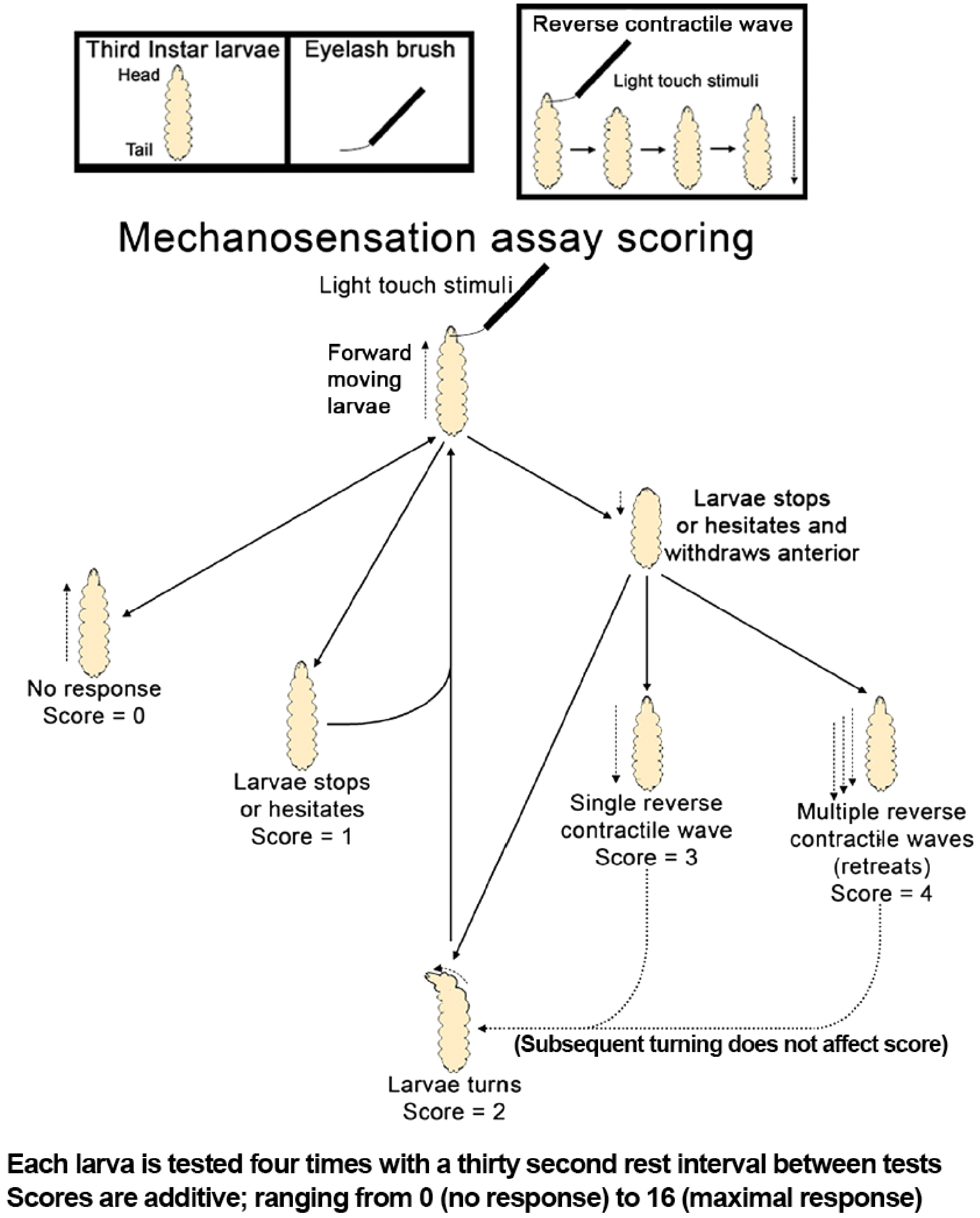

Figure S7. Larval touch sensitivity assay. Modified from (Kernan et al., 1994).

Biology Open • Supplementary information

Touch response score distribution

A

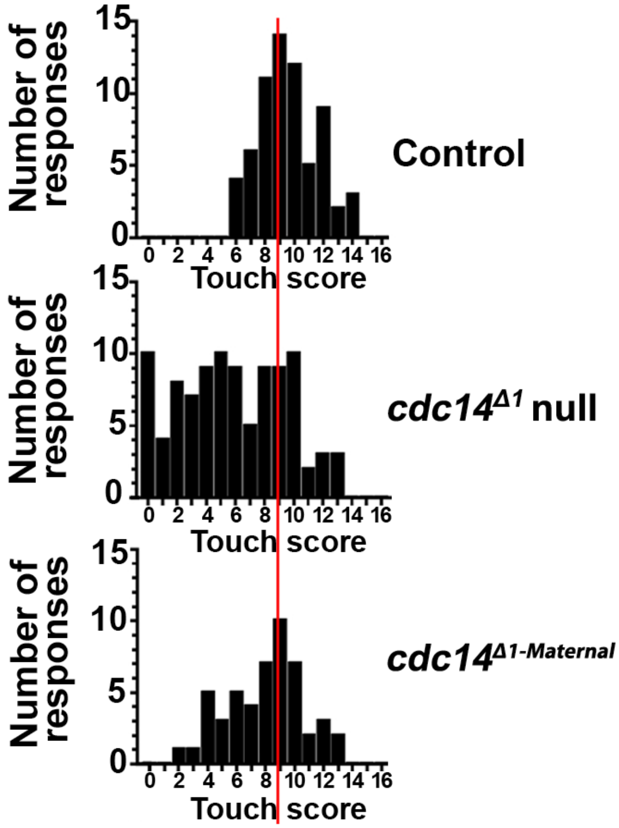

B

Genotype	Median	Standard deviation
Control	9	2.0
cdc14^{Δ1} null	6	3.6
cdc14$^{Δ1\text{-Maternal}}$	8	2.6

Figure S8. *cdc14* null larvae exhibit decreased sensitivity to mechanical stimuli. (A) Distribution of touch response scores (see Figure S8). *cdc14*^{Δ1} null larvae with maternally contributed *cdc14* (*cdc14*$^{Δ1\text{-Maternal}}$) exhibited an approximately normal distribution of scores relative to control with a peak at 9, whereas the *cdc14* null larvae exhibited a flat distribution. The mean score for *cdc14 null* larvae was significantly lower than controls ($ρ<0.001$) and *cdc14*$^{Δ1\text{-Maternal}}$ ($ρ<0.001$). Data sets were analyzed by one-way ANOVA with Tukey HSD post hoc analysis. Red vertical line delineates the mean touch response score for control. (B) Mean touch response scores with standard deviation for control, *cdc14* null, and *cdc14*$^{Δ1\text{-Maternal}}$ larvae. Results were aggregated from N≥50 larvae in N≥5 repetitions.

Biology Open • Supplementary information

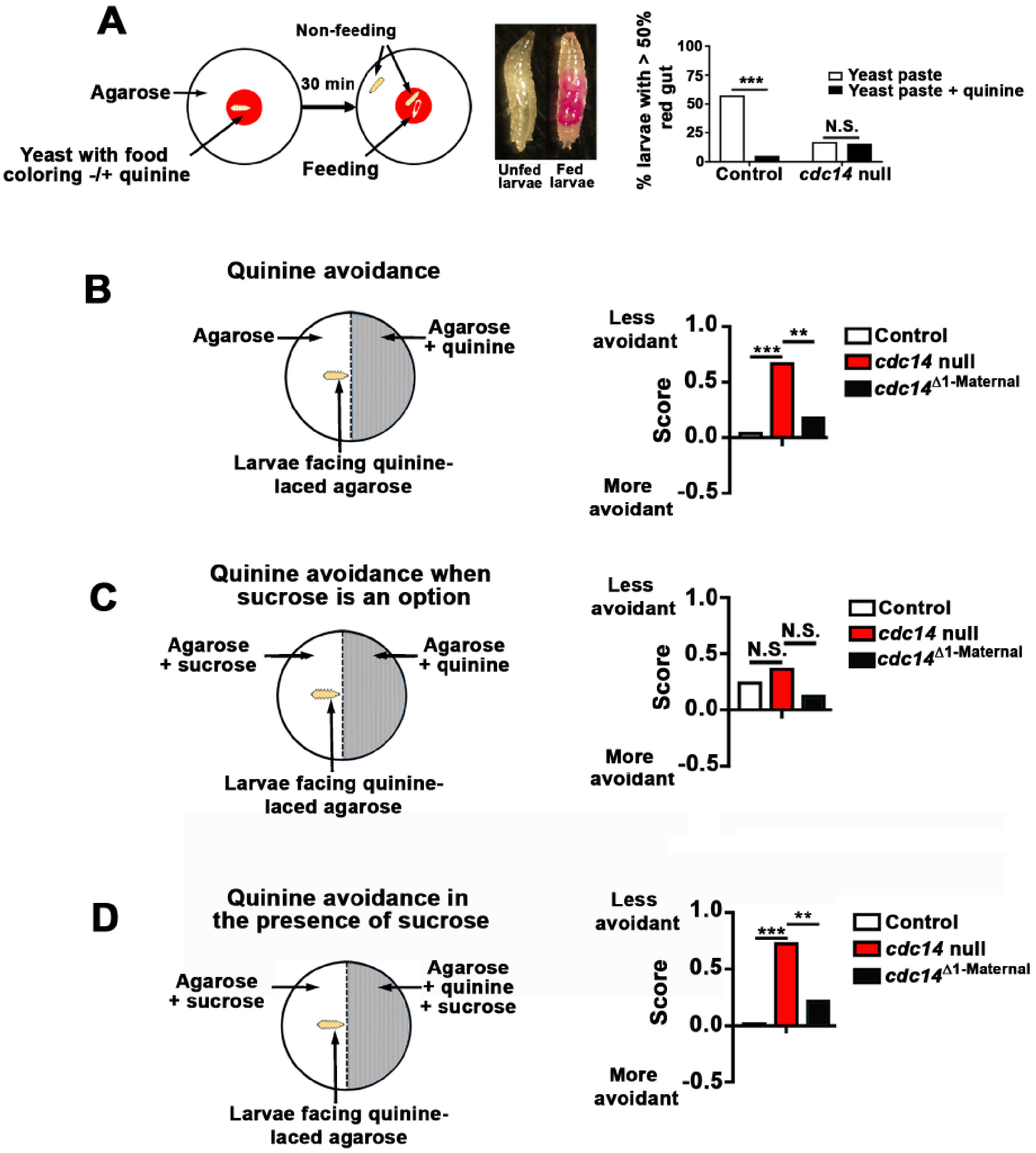

Figure S9. *cdc* null larvae show decreased but indiscriminate feeding and loss of chemosensory responses. (A) Diagram of yeast feeding assay (left). *cdc14* null larvae

Biology Open • Supplementary information

fed at the same rate in the absence or presence of quinine in contrast to controls (right). Furthermore, in the absence of quinine, *cdc14* null larvae fed at a significantly lower rate than control larvae (***). Data were aggregated from N≥40 larvae in N≥3 repetitions. Data was analyzed by Chi-squared test with Bonferroni correction; ** $\rho<0.004$, *** $\rho<0.0004$. All possible comparisons were made. (B), (C), and (D) Diagrams of quinine preference assays used are shown to the left. (B) *cdc14* null larvae showed reduced avoidance of quinine. (C) No difference was observed between *cdc14* null and control larvae when sucrose was present in one-half of the plate and quinine present in the other half. (D) *cdc14* null larvae did not avoid quinine in the presence of sucrose. Results were aggregated from N≥51 larvae in N≥15 replicates and analyzed by Chi-squared test with Bonferroni correction in (B), (C), and (D). Control larvae were compared to *cdc14* null and $cdc14^{\Delta 1\text{-}Maternal}$. *cdc14* null larvae were also compared to $cdc14^{\Delta 1\text{-}Maternal}$. ** $\rho<0.004$, *** $\rho<0.0004$, N.S. = not significant.

Biology Open • Supplementary information

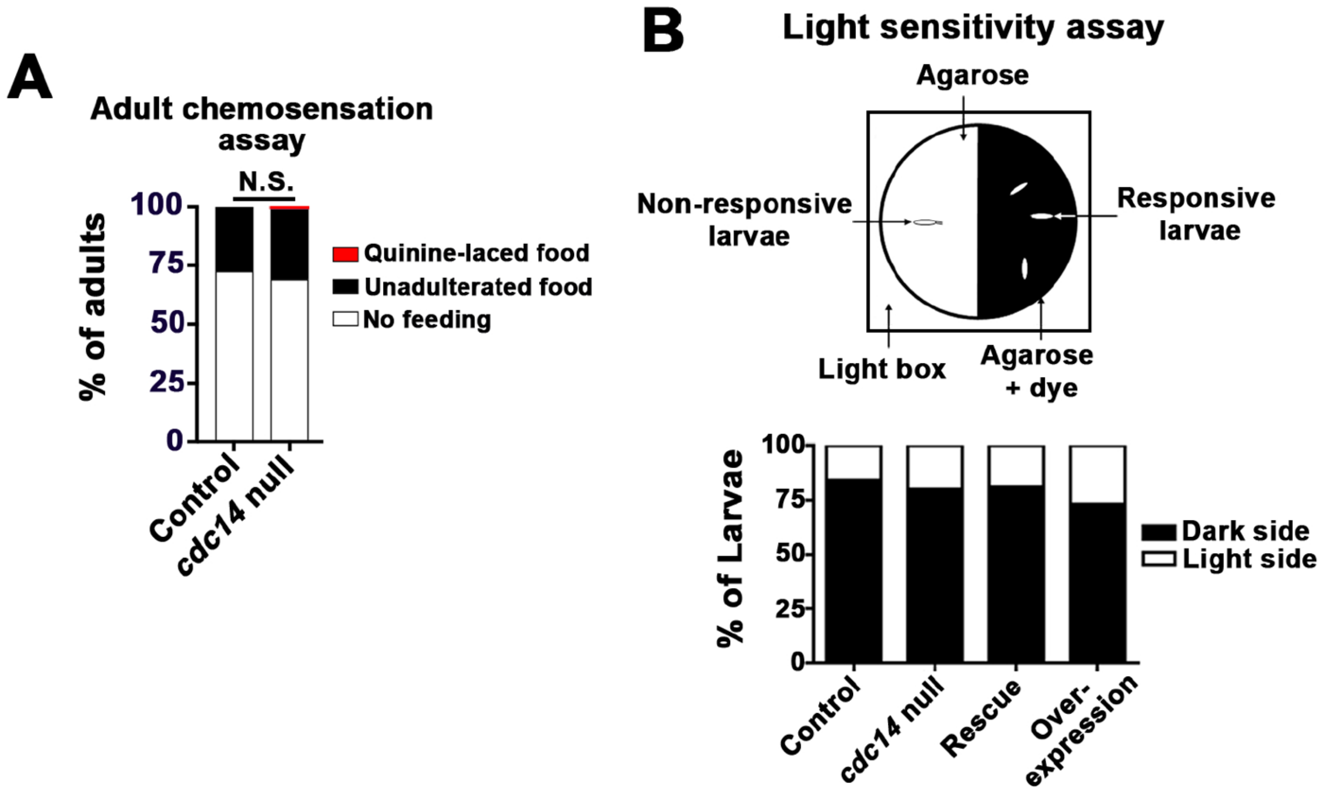

Figure S10. Adult chemosensation and larval phototaxis is unaffected by loss of *cdc14*. (A) Adult flies (three days old) were starved for 18 h, transferred to the dark, and given the choice between regular versus quinine-laced food. Flies were allowed to feed for 90 minutes, frozen, and assessed for feeding. No difference in the rate of feeding or preference for quinine was observed in adult flies. N≥423 animals from N=6 replicates were tested. (B) Diagram of light sensitivity assay (top). Light sensitivity was not significantly different between any groups. Results were aggregated from N≥100 larvae in N=3 repetitions. Results shown in (A) and (B) were analyzed by Chi-squared test with Bonferroni correction.

Biology Open • Supplementary information

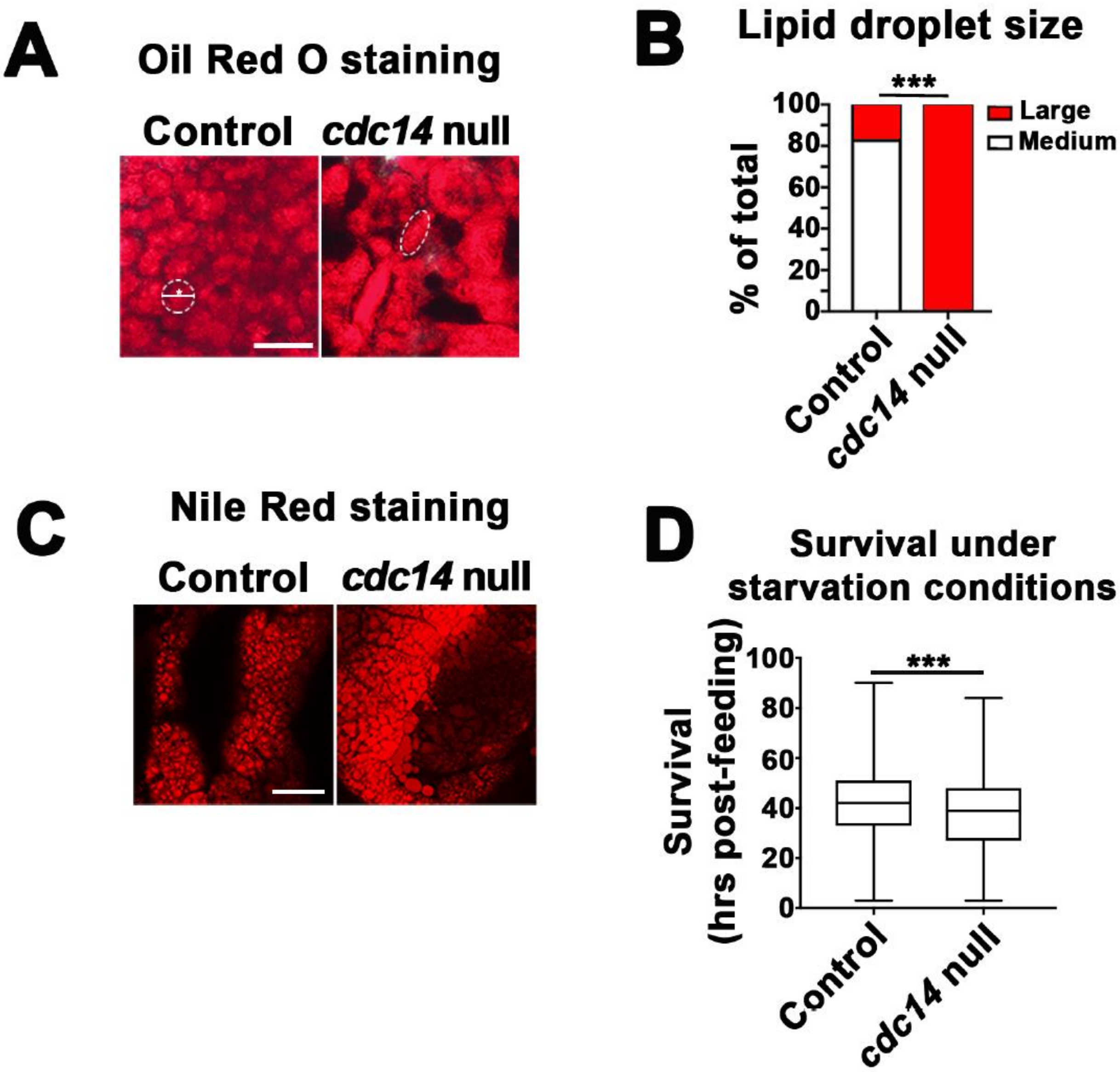

Figure S11. *cdc14* is required for normal lipid metabolism and survival during starvation conditions. (A) Oil Red O stained anterior fat bodies from segments T3 and A1 of late third instar larvae. Lipid droplets in fat bodies of *cdc14* null larvae were larger and more irregular in shape compared to controls. A regular shape droplet is outlined by a dotted white line in the control, and an irregular droplet is outlined in the null. Scale bar, 50 μm. N≥5 pelts were assessed. (B) Quantification of Oil Red O stained fat body cross-sections. *cdc14* null larvae lipid droplets were significantly larger than that of

Biology Open • Supplementary information

control larvae. Droplets from pelts were measured across their widest point; see asterisk (*) in (A). Droplets >2 standard deviations larger (≥160 μm) than the average droplet were classified as large. Samples were scored blind. N≥60 droplets were assessed from N≥5 pelts per genotype. Fisher's exact test was used to determine significance. ***ρ<0.001. (C) Larval fat bodies from the inner surface of the cuticle of abdominal segment A2-A4 stained with Nile Red. Large and irregularly shaped lipid droplets were observed in *cdc14* null larvae. Scale bar, 50 μm. N≥5 pelts were assessed per genotype. (D) Resistance to starvation of *cdc14* null and control adult females. Box and whisker plot shows that control animals lived modestly longer (> 4.25 h) than the *cdc14* null adult. Results were aggregated from N≥347 animals in N≥15 experiments using a t-test (two tailed, equal variance). *** ρ<.001.

Biology Open • Supplementary information
